# High voltinism, late-emerging butterflies are sensitive to interannual variation in spring temperature in North Carolina

**DOI:** 10.1093/ee/nvae110

**Published:** 2024-11-07

**Authors:** Laura E Hamon, Joel G Kingsolver, Kati J Moore, Allen H Hurlbert

**Affiliations:** Department of Entomology & Plant Pathology, North Carolina State University, Raleigh, NC, USA; Department of Biology, The University of North Carolina at Chapel Hill, Chapel Hill, NC, USA; Department of Biology, Duke University, Durham, NC, USA; Department of Biology, The University of North Carolina at Chapel Hill, Chapel Hill, NC, USA; Environment, Ecology and Energy Program, University of North Carolina, Chapel Hill, NC, USA

**Keywords:** phenology, climate change, butterflies, Southeastern United States, voltinism

## Abstract

Climate change has been repeatedly linked to phenological shifts in many taxa, but the factors that drive variation in phenological sensitivity remain unclear. For example, relatively little is known about phenological responses in areas that have not exhibited a consistent warming trend, making it difficult to project phenological responses in response to future climate scenarios for these regions. We used an extensive community science dataset to examine changes in the adult flight onset dates of 38 butterfly species with interannual variation in spring temperatures in the Piedmont region of North Carolina, a region that did not experience a significant overall warming trend in the second half of the 20th century. We also explored whether voltinism, overwintering stage, and mean adult flight onset dates explain interspecific variation in phenological sensitivity to spring temperature. We found that 12 out of 38 species exhibited a significant advance in adult flight onset dates with higher spring temperatures. In comparison, none of the 38 species exhibited a significant advance with year. There was a significant interaction between mean onset flight date and voltinism, such that late-emerging, multivoltine species tended to be the most sensitive to spring temperature changes. We did not observe a significant correlation between phenological sensitivity and the overwintering stage. These results suggest that butterfly arrival dates may shift as temperatures are projected to rise in the southeastern United States, with late-emerging, multivoltine species potentially exhibiting the greatest shifts in adult flight onset dates.

## Graphical Abstract

**Graphical Abstract:**
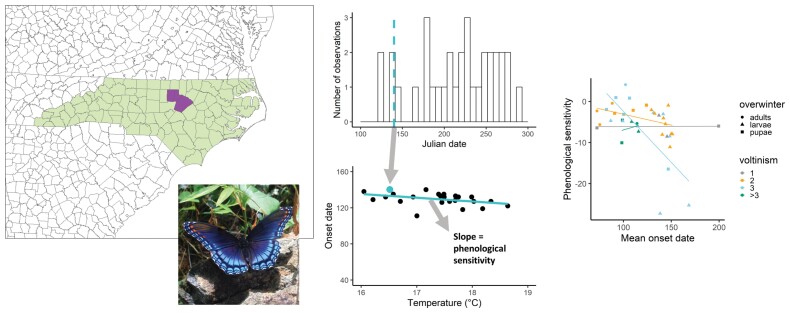

## Introduction

Mounting evidence indicates that species are undergoing significant changes in seasonal timing and distribution on a global scale in response to climatic change, especially at higher latitudes (Walther et al. 2002, Parmesan and Yohe 2003). These patterns have repercussions for individual fitness, community interactions, and the continued persistence of sensitive species (Møller et al. 2008, Iler et al. 2021, Colom et al. 2022). Therefore, as global temperatures rise, it is increasingly crucial to study the consequences of changes in temperature on seasonal timing and biological processes.

Appearance date is an informative measure for examining how seasonally active species respond to changing temperatures and is consequently used in myriad studies of climate change response (Parmesan 2007, Parmesan et al. 2022). Butterflies are a popular model organism for studying changes in phenology because they have predictable and readily observable life events (Roy et al. 2001) and are ectothermic, making them sensitive to changes in temperature (Dennis 1993). Additionally, butterflies have long been a popular subject for naturalists and hobbyists, which has allowed for the persistence of long-term datasets of butterfly observations and museum specimens (Thomas 2005, Eskildsen et al. 2015, Prudic et al. 2017). As temperatures have increased, adult flight onset dates (hereafter onset dates) have advanced in many butterfly species, as observed in California (Forister and Shapiro 2003), the Mediterranean Basin (Stefanescu et al. 2003), the United Kingdom (Roy and Sparks 2000, Diamond et al. 2011), and Ohio (Diamond et al. 2014).

While there have been multiple studies of changes in butterfly onset dates from around the globe (e.g., Stefanescu et al. 2003, Diamond et al. 2011, Zografou et al. 2021), our understanding of this phenomenon in the southeastern United States and other humid subtropical regions is limited. This region is noteworthy because it has not experienced the same clear upward trend in temperature as observed on a global scale. Rather, the southeastern United States experienced a slight cooling trend over the latter half of the 20th century (Portmann et al. 2009) albeit with substantial interannual variation (Supplementary Fig. S1). This designates the southeastern United States as a climatically unique region with the potential to shed light on future phenological shifts as temperatures are projected to rise (Carter et al. 2014). Previous studies in the Southeast have observed shifts in flowering time in years with higher temperatures (Park and Schwartz 2014, Pearson 2019), demonstrating that phenological change is likely as temperatures continue to rise. However, to our knowledge, adult butterfly phenology has not been studied for the southeastern United States specifically.

Phenological shifts are variable across taxa as well as across geographic space. Species traits have been shown to play a role in determining phenological sensitivity as measured by phenological shifts in response to temperature, time, and their interaction (Diamond et al. 2011, Karlsson 2014, Larsen et al. 2022). However, the strength and direction of the effects of species traits on phenological sensitivity can vary between geographic regions and species (Walther et al. 2002). For example, several studies suggest that the life stage at which overwintering occurs can be an effective predictor of phenological sensitivity (Diamond et al. 2011, Kharouba et al. 2014, Larsen at al. 2024), while several other studies have observed minimal to no influence of overwintering stage (Forister and Shapiro 2003, Brooks et al. 2017). In addition, certain traits, such as whether a species tends to appear earlier or later in the year, have seldom been studied directly (see Karlsson 2014, Kharouba et al. 2014). Given that species with limited phenological sensitivity can experience declines in abundance (Colom et al. 2022, Larsen et al. 2024), it is crucial to determine how ecologically relevant species traits affect phenological response, and how these factors may vary between geographic areas.

In this study, we use a database of butterfly observations collected opportunistically by community scientists in Durham, Orange, and Wake Counties of North Carolina to examine the role of spring temperature in affecting onset dates in this region from 1993 to 2020 (Supplementary Fig. S1). We also explore how species-specific traits relate to changes in onset dates in North Carolina butterflies, focusing on voltinism, overwintering stage, and average onset dates. We predict that onset dates advance with higher spring temperatures, reflecting the trend demonstrated by studies in other regions (e.g., Stefanescu et al. 2003, Diamond et al. 2011, Zografou et al. 2021). For the same reason, we also expect that species in this region with high voltinism, advanced overwintering stages, and early average onset dates will exhibit greater phenological sensitivity compared to those with lower voltinism, early overwintering stages, and late-onset dates.

## Methods

### Study System

We used a dataset of opportunistic butterfly observations maintained by the North Carolina Division of Parks and Recreation. This dataset is largely comprised of records submitted to an email listserv that connects butterfly enthusiasts throughout North Carolina. This dataset also incorporates museum specimens, North American Butterfly Association counts, Bioblitzes, and a limited subset of iNaturalist records, particularly for rare species, meaning that it includes a variety of approaches to opportunistic data collection. These data are hosted and maintained by the North Carolina Biodiversity Project and North Carolina State Parks (LeGrand and Howard 2022). The database was launched in 1993 and covers North Carolina’s 177 known butterfly species. It is updated yearly and, at the time of this study, includes at least 232,779 records from 1899 to 2020. Each entry lists the common name, date, observer name, number of individual butterflies observed, and county.

We selected observations from the North Carolina Triangle region (Durham, Orange, and Wake Counties, Supplementary Fig. S1) since these counties had the highest and most consistent sampling effort. In addition, Durham, Orange, and Wake Counties are all within the Piedmont ecoregion and have similar climatic conditions. We selected records collected between 1993 and 2020 because there are few records prior to this interval. Out of these years, we selected species that had at least 10 years with at least 10 unique observer dates per year and excluded species that are migratory in the Piedmont ecoregion. We treated *Erynnis horatius* and *E. juvenalis* as a single taxon, *Erynnis* spp., since observers frequently consider time of year to distinguish these very similar species, rendering it difficult to meaningfully interpret any differences in phenology. For the same reason, we also treated *Celastrina ladon* and *Celastrina neglecta* as a single taxon, *Celastrina* spp. We included a total of 38 species or taxa (hereafter referred to as species) in our analysis, representing 33,251 records (Supplementary Table S1). We used R (v4.1.1; R Core Team 2021) for all analyses.

### Adult Flight Onset Date

**Fig. 1.**
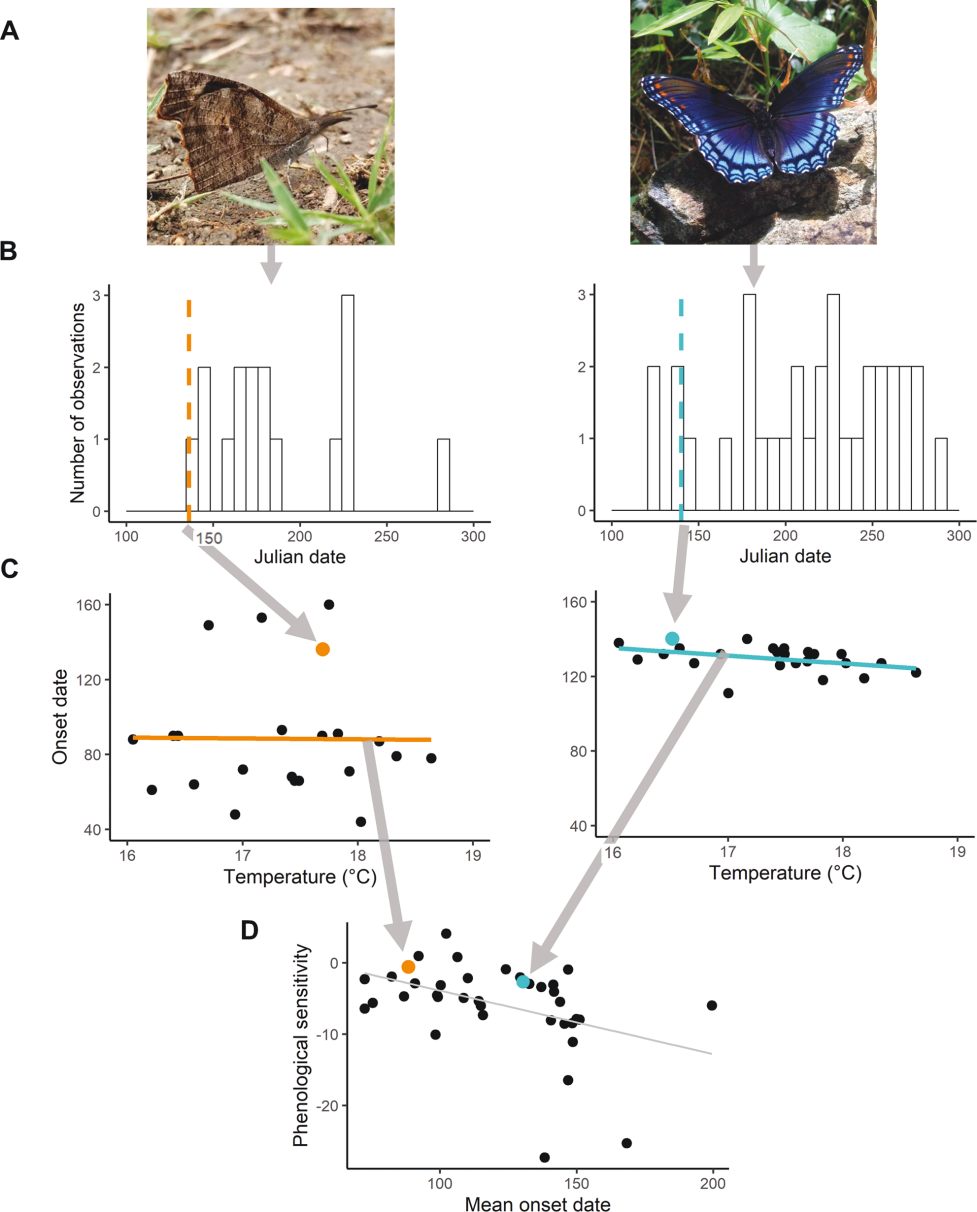
Methods for determining phenological sensitivity (days/°C) using A) 2 example focal species, American snout (Libytheana carinenta), and red-spotted purple (Limenitis arthemis astyanax). Values for each species are indicated with orange and light blue symbols, respectively. B) Histograms of records by Julian date collected for both species in 2015 and 1996, respectively. Vertical dashed lines indicate calculated onset date (the Julian date on which 10% of records for that year were collected) for each species. C) Regression plots of onset date versus temperature, with the calculated onset date indicated with a large, colored point. D) Phenological sensitivity vs. mean appearance date plot, with onset date vs. temperature slopes marked with large, colored points.

Since the first record of appearance is heavily influenced by outliers (van Strien et al. 2008), we calculated the onset date as the date on which 10% of records had been collected for each unique species-year (Fig. 1A,B). For a given species, we only calculated the onset date for years with at least 10 unique observer dates.

We ignored information on abundance and defined a “record” as each unique combination of observer, date, county, and species in order to limit the outsized influence of survey efforts such as BioBlitzes and North American Butterfly Association butterfly counts (https://www.naba.org), whose large tallies greatly skewed perceived onset dates. Thus, a single observer reporting 6 individuals of a given species on a particular date would only count as a single record, whereas 2 separate observers who each reported 1 individual from each of the 3 counties represent 6 records.

### Spring Temperature

Mean monthly temperature data was obtained from the PRISM Climate Group (Oregon State University). We used the packages “raster” (Hijmans 2021) and “rgdal” (Bivand et al. 2021) to subset spatial temperature data from Durham, Orange, and Wake Counties. For a given year, we defined the spring temperature as the mean monthly temperature for each county averaged over a static 4-mo window that encompasses spring conditions (March to June) and then averaged these values across the 3 counties. We elect to use a fixed window to determine the effect of spring temperatures on the variation in the timing of butterfly emergence in this system.

### Phenological Response

For each species, we fit linear regression models to determine how the onset date varied as a function of either spring temperature or year (Fig. 1C). When observing the distribution of onset dates, we noted that some estimates differed from the average onset date for a species by a wide margin (e.g., over 3 mo). Since our dataset incorporates a wide variety of data sources collected over a non-standardized temporal window, it is possible that some estimates are still subject to influences that are not biologically relevant, such as changes in observer activity. We therefore elected to omit outliers from our linear regression models by identifying and removing highly influential data points. To identify highly influential data points, we calculated Cook’s distance, which is a measure of the relative influence of a data point on a model outcome (Cook 1977). We then excluded points where Cook’s distance was greater than 4 divided by the total number of data points for each species. We used this cutoff point because is frequently used as the standard for determining outliers using Cook’s distance (e.g., Altman and Krzywinski 2016) but also because it conservatively identified possible outlier data points. For comparison, a description of methods with outliers included is outlined in Supplementary Appendix S1.

For each species and predictor (spring temperature or year), we then calculated the slope, the mean onset date, and the standard deviation of the onset date. We used the slope of the fitted onset date-temperature relationship as an estimate of phenological sensitivity (days/°C) to average spring temperature.

### Species Traits

To examine whether species traits explain variation in phenological sensitivity, we considered voltinism, overwintering stage, and mean onset date of each species as predictors. We treated voltinism as a continuous variable and again used values that were specific to the Piedmont region of NC. Voltinism for butterfly species in this region ranges between one generation per year to 4 or more generations per year. In cases where a species had variable voltinism, we selected the average value. For example, if a species was reported to have 3–4 generations per year, we assigned a value of 3.5. We treated the overwintering stage as a factor (larvae, pupae, or adults) and when geographically variable, used the reported overwintering stage specific to the southeastern United States, especially the Piedmont region of NC. Mean onset date was treated as a continuous estimate of whether the first generation of each species appears earlier or later in the year (Table 1, Fig. 1D). Given the small sample size of 38 species, we also examined the distribution of mean onset date by overwintering stage, since these traits potentially covary. If the mean onset date and overwintering stage did indeed covary, we elected to include the more parsimonious, continuous measure of the mean onset date. Voltinism and overwintering stage were referenced chiefly from LeGrand and Howard (2022), but other sources include Butterflies and Moths of North America (https://www.butterfliesandmoths.org) and the University of Florida Department of Entomology and Nematology (https://www.entnemdept.ufl.edu/) (see Supplementary Table S1). Although the overwintering stage can be associated with voltinism in Northern European species (Teder 2020), we did not observe such a link in our focal species.

**Table 1.** Summary of slopes, *r*^2^, *P*-values, and sample sizes per species from linear regression models of onset date vs. spring temperature in NC Triangle butterfly species, in addition to the mean and standard deviation of onset date for each species

Species	Slope	*R*-squared	*P* value	Mean onset date	Standard deviation onset date	Number of records
*Abaeis nicippe*	4.10	0.01	0.68	102.24	32.29	1,362
*Ancyloxypha numitor*	−8.47	0.09	0.20	148.40	20.99	563
*Anthocharis midea*	−6.43	0.63	<0.001	72.41	5.49	448
*Asterocampa celtis*	−8.06	0.37	<0.01	140.57	9.61	414
*Atalopedes campestris*	−25.28	0.38	<0.01	168.37	29.16	1,176
*Battus philenor*	0.82	<0.01	0.87	106.37	12.62	458
*Calycopis cecrops*	−2.06	<0.01	0.85	129.30	38.08	577
*Celastrina spp.*	−1.95	0.02	0.49	82.35	9.08	1,139
*Colias eurytheme*	−4.55	0.03	0.41	98.93	19.98	922
*Cupido comyntas*	−4.91	0.12	0.09	108.62	9.76	1,728
*Cyllopsis gemma*	−4.77	0.09	0.22	99.26	9.96	405
*Epargyreus clarus*	−0.90	<0.01	0.83	124.08	13.38	1,431
*Erynnis spp.*	−4.70	0.18	0.03	86.80	7.20	1,274
*Euphyes vestris*	−7.89	0.18	0.06	149.95	13.47	392
*Eurytides marcellus*	0.95	0.01	0.74	92.12	8.31	282
*Hermeuptychia sosybius*	−6.02	0.15	0.05	114.96	10.51	1,119
*Lerema accius*	−5.99	0.03	0.45	199.48	24.58	1,012
*Lethe anthedon*	−11.09	0.39	0.02	148.62	13.59	207
*Lethe appalachia*	−4.07	0.05	0.51	141.75	14.71	213
*Libytheana carinenta*	−0.45	<0.001	0.97	88.36	32.67	550
*Limenitis archippus*	−3.05	0.01	0.62	141.50	19.97	421
*Limenitis arthemis*	−2.75	0.04	0.33	131.35	9.91	1,161
*Megisto cymela*	−3.39	0.31	0.03	137.06	4.11	241
*Papilio glaucus*	−2.87	0.04	0.31	90.86	10.33	2,205
*Papilio polyxenes*	−3.13	0.04	0.35	100.30	10.40	580
*Papilio troilus*	−2.15	0.01	0.63	110.19	13.91	824

Species	Slope	*R*-squared	*P* value	Mean onset date	Standard deviation onset date	Number of records
*Phyciodes tharos*	−7.32	0.19	0.03	115.76	11.08	1,967
*Pieris rapae*	−10.06	0.10	0.12	98.39	22.72	1,092
*Polites origenes*	−0.94	<0.01	0.82	146.90	10.62	343
*Polygonia comma*	−2.30	0.01	0.65	72.43	14.38	549
*Polygonia interrogationis*	−5.63	0.06	0.21	75.35	15.30	982
*Pompeius verna*	−5.49	0.26	0.02	143.95	8.18	454
*Pyrgus communis*	−27.29	0.20	0.03	138.29	44.40	714
*Speyeria cybele*	−8.52	0.33	0.03	145.57	9.42	217
*Strymon melinus*	−16.47	0.12	0.09	146.88	29.79	726
*Thorybes bathyllus*	−2.94	0.05	0.47	132.69	9.29	175
*Vanessa virginiensis*	−5.35	0.06	0.26	114.20	15.34	1,437
*Wallengrenia otho*	−7.94	0.53	<0.01	151.13	8.71	240

We examined variations in phenological sensitivity between species with different traits. To do this, we ran separate models for both spring temperature and year. In all models, we inversely weighted the values by the standard deviation of the onset date (Table 1) so that species with extremely variable onset date had less influence in the model since these estimates are likely to be less precise. Linear models were summarized using the “Anova” function within the package “car” using a Type III sums of squares where interactions were significant (v.3.0.11, Fox and Weisberg 2019). Where appropriate, we compared simple and complex models using an ANOVA.

To test for a phylogenetic signal in our findings, we fit a variance component model to determine the percentage of the variance in phenological sensitivity that can be attributed to taxonomic level (family, subfamily, genus, or species).

## Results

### Phenological Response

**Fig. 2.**
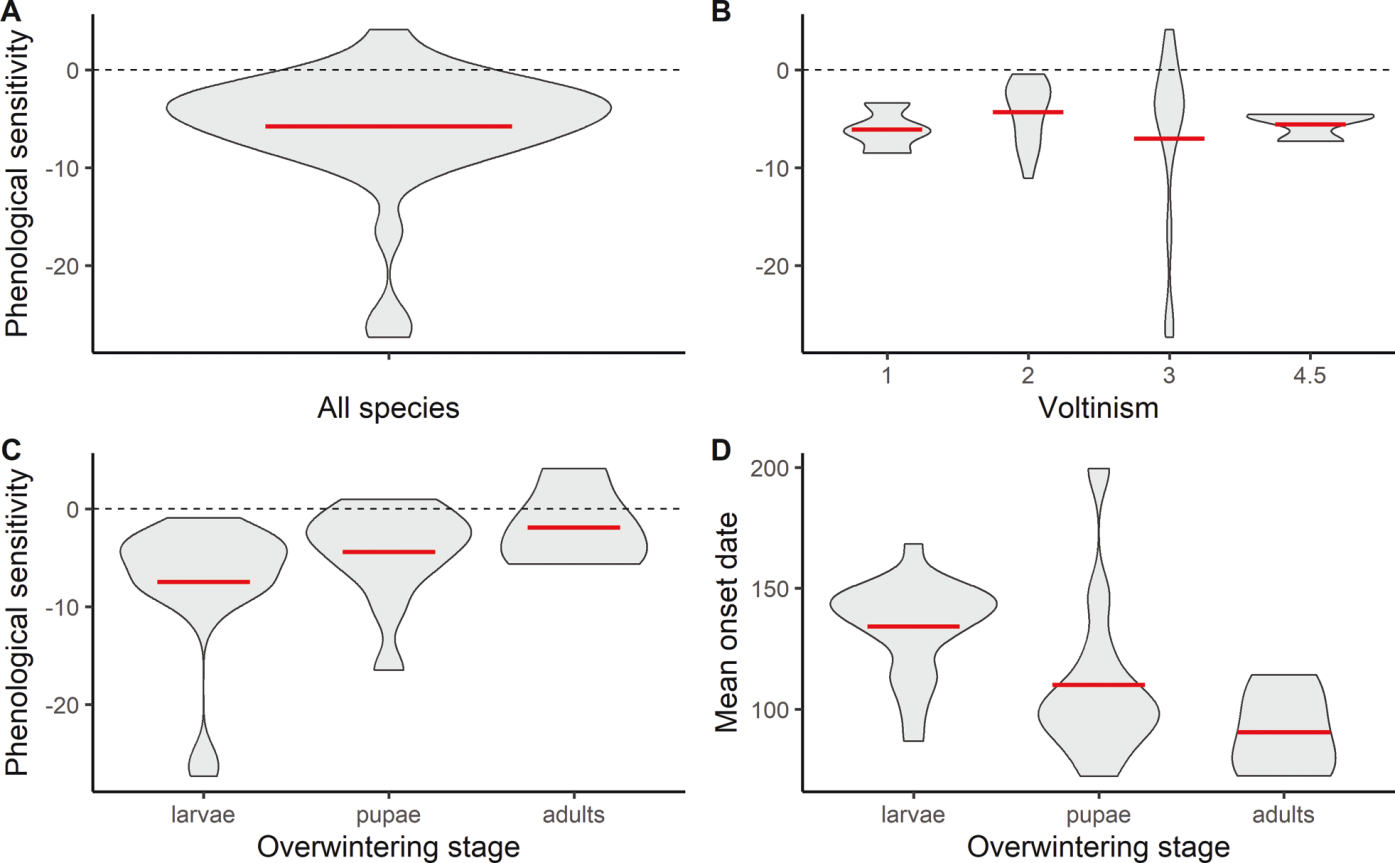
Distribution of responses. The horizontal dotted line indicates zero and the horizontal bold line indicates the mean value. A) Distribution of phenological sensitivity values (days/°C) for all focal species. B) Distribution of phenological sensitivity values by voltinism. The single species with a voltinism of 3.5 generations has been excluded here for clarity. C) Distribution of phenological sensitivity values by overwintering stage. D) Distribution of average onset date by overwintering stage.

Of the 38 focal species, 35 exhibited earlier onset dates in years with warmer springs (Fig. 2A, all onset date vs. temperature plots in Supplementary Appendix S2), with 12 species exhibiting significant (*P* < 0.05) negative slopes (Table 1). The median response was for a species to appear 4.7 days earlier for every 1°C increase in average spring temperature, with some species shifting up to 27 days/°C (Table 1). When examining year as the explanatory variable, just 2 of the 38 focal species exhibited a significant relationship (*P* < 0.05), both of which were positive (i.e., later onset date with increasing year) (Supplementary Table S2).

### Species Traits

When examining voltinism alone, we did not observe a significant difference in phenological sensitivity between butterflies of differing voltinism (*F*_1,36_ = 0.04, *R*^2^ = −0.027, *P* = 0.841, Fig. 2B). When we examined the distribution of phenological sensitivity by overwintering stage (Fig. 2C) and compared this to the distribution of mean onset date by overwintering stage, we observed a pattern in which species that overwinter as larvae tend to have later mean onset dates and those that overwinter as adults tend to have earlier mean onset dates (Fig. 2D), such that mean onset date may partially serves as a proxy for overwintering stage. Therefore, we elected to interpret a more parsimonious model that excluded the categorical overwintering stage in favor of the continuous estimate of the mean onset date. In addition, adding the overwintering stage did not improve the model fit when compared with a model with mean onset date, voltinism, and their interactions (ANOVA, *F*_2,32_ = 0.098, *P* = 0.907). We also did not observe a significant effect of the interaction between voltinism and overwintering stage on phenological sensitivity (*F*_2,32_ = 0.11, *P* = 0.89).

**Fig. 3.**
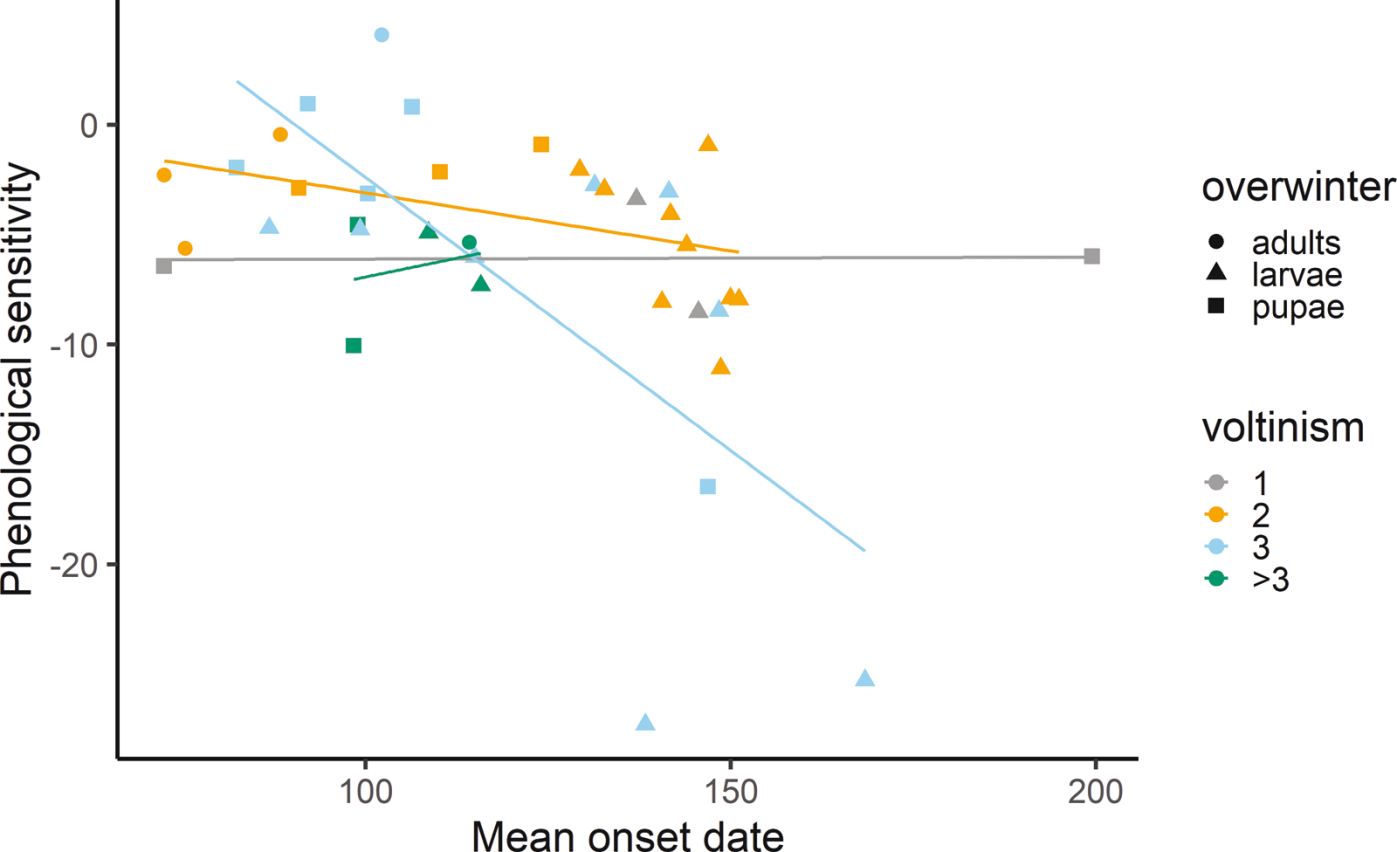
Interaction plot demonstrating phenological sensitivity (days/°C) versus mean onset date by voltinism and overwintering stage. Voltinism is treated as a factor for clarity in this figure. Regression lines corresponding to each voltinism value are included to visualize the interaction. Circles, triangles, and squares indicate species that overwinter as adults, larvae, and pupae, respectively.

There was an interaction between voltinism and mean onset date (*F*_1,34_ = 5.84, *P* = 0.021 Fig. 3), such that the relationship between phenological sensitivity and mean first appearance was stronger among species with higher voltinism compared to those that only have a single brood per year (Fig. 3). The model that included the interaction term between voltinism and mean onset date (*F*_3,34_ = 4.055, *R*^2^ = 0.199, *P* = 0.014) was a better fit than the model with voltinism alone (*F*_1,36_ = 0.036, *R*^2^ = −0.027, *P* = 0.851; model comparison: ANOVA, *F*_2,34_ = 6.06, *P* = 0.006). In addition, the model that included voltinism and mean onset date as an interaction term was a better fit than the model with the additive effects alone (*F*_2,35_ = 2.777, *R*^2^ = 0.088, *P* = 0.076; model comparison: ANOVA, *F*_1,34_ = 5.84, *P* = 0.02). When examining the influence of taxonomic level on phenological sensitivity, we found that 80% of the variance is explained at the level of genus or below, while family and subfamily explain less than 5% (Supplementary Fig. S2). Given that our 38 species span 33 different genera, it is likely that variation in phenological sensitivity is not driven by phylogenetic relatedness.

### Analysis with Outliers Included

For comparison, see Supplementary Appendix 1 for a description of results with outliers included (Supplementary Figs. S3–S5, Supplementary Tables S3–S4, with all onset date vs. temperature plots in Supplementary Appendix S3).

## Discussion

The observed relationship between onset date and spring temperatures in the Piedmont region of North Carolina is consistent with trends demonstrated by previous studies using long-term butterfly data (Roy and Sparks 2000, Forister and Shapiro 2003, Stefanescu et al. 2003, Diamond et al. 2011, Larsen et al. 2024), and with a well-established trend of the influence of rising temperatures on the phenology of ectothermic species (Forister and Shapiro 2003, Parmesan and Yohe 2003). Specifically, we observed a mean advancement of 4.7 days for every 1°C increase in temperature, compared to average advancements of 2.4 days in Canadian butterflies (Kharouba et al. 2014) and 2–10 days in UK butterflies (Roy and Sparks 2000). Butterflies are highly sensitive to changes in temperature (Pollard et al. 1988, Dennis 1993), and spring climatic conditions play a role in determining the development rate of early overwintering stages, as well as the ability for adults to be active (Dell et al. 2005). Though other climatic variables such as winter temperatures (Forister and Shapiro 2003) and precipitation (Crossley et al. 2021) may also play a role in this system, the observed results confirm that butterflies in the Piedmont region of NC shift the timing of adult flight onset in response to year-to-year variation in spring temperature in a manner consistent with phenological responses observed in regions with significant rising temperature trends.

We observed that species with both high voltinism and late mean onset dates were more sensitive to spring temperatures, but the reason for this trend is not immediately clear. Regarding voltinism alone, previous studies have demonstrated variable results concerning the strength and direction of voltinism on phenological sensitivity (Kharouba et al. 2014, Zografou et al. 2021, Larsen et al. 2022). Zougrafou et al. (2021) observed that species with high voltinism were more sensitive to changes in temperature. They suggested that species with high voltinism may be more responsive to climatic variability (Roy et al. 2015), reflecting their greater ability to vary the number of generations in a given year (Tobin et al. 2008, Altermatt 2010). Conversely, Larsen et al. (2022) observed no significant correlation between changing phenology and voltinism. It is worth noting that voltinism can increase with climatic warming (Tobin et al. 2008, Altermatt 2010), which points to the fact that voltinism is apparently both a trait that varies between species, as well as a plastic life history strategy that varies across years and populations (e.g., Lindestad et al. 2019). The relationship between phenology and voltinism could even have implications for the long-term survival of certain species. A study on UK butterflies observed that univoltine species with advanced phenology in warmer years were more likely to have a decline in population compared to multivoltine species (Macgregor et al. 2019). To understand why a subset of species with high voltinism exhibited greater phenological sensitivity in our system, it may be necessary to disentangle the proximate explanations for how voltinism varies with temperature. In other words, are high-voltinism species more sensitive to temperature, or do species that are more sensitive to temperature tend to exhibit high voltinism? Voltinism may indeed reflect underlying linked traits that are otherwise not considered in our models (Levy et al. 2015, Teder 2020).

In contrast to findings from several previous studies at higher latitudes (Hassall et al. 2007, Diamond et al. 2011, Kharouba et al. 2014, Brooks et al. 2017), we observed that late-emerging species were broadly more sensitive to variation in average spring temperature compared to early-emerging species of similar voltinism. One possible explanation is that late-emerging species may be experiencing higher temperatures during a longer developmental window (i.e., during the larval or pupal stage), and changes in average temperature over this extended window may dramatically affect their emergence time. Thermal accumulation—typically measured as growing degree days—has previously been shown to be an effective predictor of emergence phenology in Ohio butterflies (Cayton et al. 2015). Given that we observed a trend wherein species that overwinter as larvae tend to have later mean onset dates (Fig. 2D), it is possible that the duration of high temperatures on developmental timing may play a role in this pattern. This result is broadly consistent with a recent study across eastern North American butterflies by Larsen et al. (2024) showing that species overwintering as eggs, and hence with later flight periods, were more phenologically sensitive than species with more developed overwintering stages. However, we found that overwintering stage did not explain significant variation in phenological sensitivity above and beyond mean onset date, perhaps in part because none of the North Carolina species examined overwinter as eggs, and the species examined here therefore represent a narrower breadth of overwintering strategies. It is also worth pointing out that we utilized a static temperature window (March—June) and therefore, late- and early-emerging species are at different life stages at a given time during this focal window. It is possible that this window may reflect a key developmental period for our late-emerging species that is highly impacted by temperature, but which is also somewhat decoupled from overwintering stage.

A key consideration when assessing our findings is that this study utilizes an opportunistic community science dataset from various sources. Opportunistic datasets present an opportunity to study trends at a geographic scale that is seldom possible with formal surveys (e.g., Soroye et al. 2018), and multiple studies have consequently leveraged opportunistic data to study phenological changes in butterflies (e.g., Karlsson 2014, Brooks et al. 2017, Larsen et al. 2022). However, opportunistic data do not account for sampling effort, meaning that records may be biased to a particular time or place (Geldmann et al. 2016). For example, we observed that interannual estimates of emergence date varied widely in our dataset, necessitating the omission of outliers. We ultimately omitted 60 species-year combinations from our analyses, pointing to the potential influence of years with unusual survey events. Furthermore, community scientists may vary in their ability to detect and identify species (Kelling et al. 2015), making it difficult to understand trends in rare or cryptic species. For this reason, we limited our analysis to just abundant species, potentially overlooking rare species that may differ in their sensitivity to warmer springs. Formal surveys in the region, such as the Carolinas Butterfly Monitoring Program (Pippen 2024) present an opportunity to supplement opportunistic datasets with effort-based surveys for a more holistic snapshot of butterfly phenological trends (Larsen et al. 2022).

We found that most of our species exhibited a negative correlation between onset date and spring temperatures, sometimes quite dramatically. Though the southeastern US did not exhibit a consistent warming trend in the 20th century, temperatures have increased over the 21^st^ century and are projected to continue climbing (Carter et al. 2014). This analysis therefore points to the potential for continued changes in first appearance as temperatures continue to warm. This could have repercussions for survival at both the individual and species level (reviewed in Parmesan 2006, Møller et al. 2008). The southeastern United States is also projected to have more frequent or intense periods of drought (Ingram et al. 2013), which we did not include in our analyses. For a more cohesive model of predicting phenological change in butterflies, additional analyses should account for climatic variables such as precipitation and the previous year temperature in addition to spring temperature.

As temperatures rise in the southeastern United States, future studies should continue to track insect phenologies to determine if they align with projections, taking care to account for other confounding global change factors such as urbanization (Diamond et al. 2014). It remains unclear whether the predicted changes in this study will have a detrimental effect on butterfly populations in North Carolina. In addition, the ramifications of changes in butterfly populations for interacting species such as host plants and predators in the southeastern US remains poorly known. Large, consistent community science datasets like the one used in this study represent an opportunity to continue to track the abundances and phenology of organisms as climate change accelerates.

## Supplementary Data

Supplementary data are available at *Environmental Entomology* online.

nvae110_suppl_Supplementary_Figure_S1

nvae110_suppl_Supplementary_Table_S1

nvae110_suppl_Supplementary_Appendix_S1

nvae110_suppl_Supplementary_Appendix_S2

nvae110_suppl_Supplementary_Table_S2

nvae110_suppl_Supplementary_Figure_S2

nvae110_suppl_Supplementary_Figure_S3

nvae110_suppl_Supplementary_Table_S3

nvae110_suppl_Supplementary_Appendix_S3

nvae110_suppl_Supplementary_Figure_S4

nvae110_suppl_Supplementary_Figure_S5

nvae110_suppl_Supplementary_Table_S4

## Data Availability

Data and code are available via the corresponding author upon reasonable request.
