# Supplementary figures and images for: High voltinism, late-emerging butterflies are sensitive to interannual variation in spring temperature in North Carolina
Source: Environ Entomol. 2024 Nov 7;54(1):77–85. doi: 10.1093/ee/nvae110 (PMC11837338; doi:10.1093/ee/nvae110)

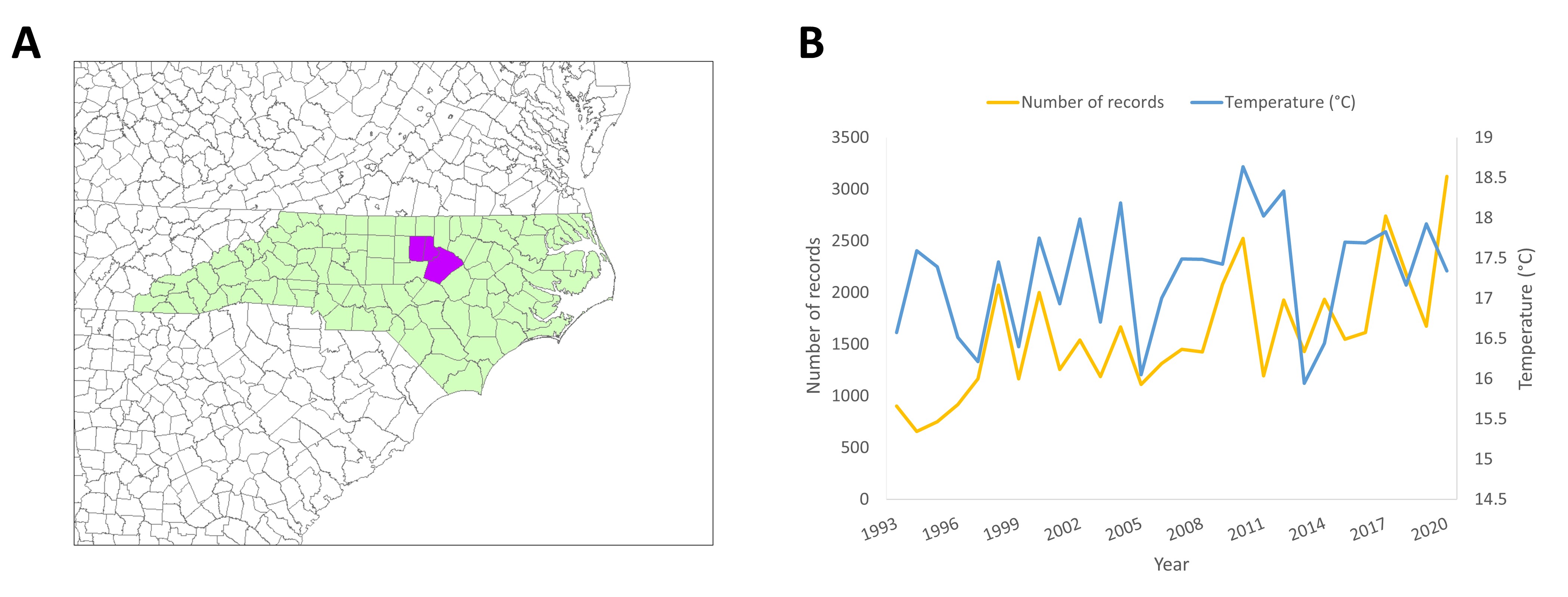

Supplement: nvae110_suppl_Supplementary_Figure_S1 [file nvae110_suppl_supplementary_figure_s1.jpeg]

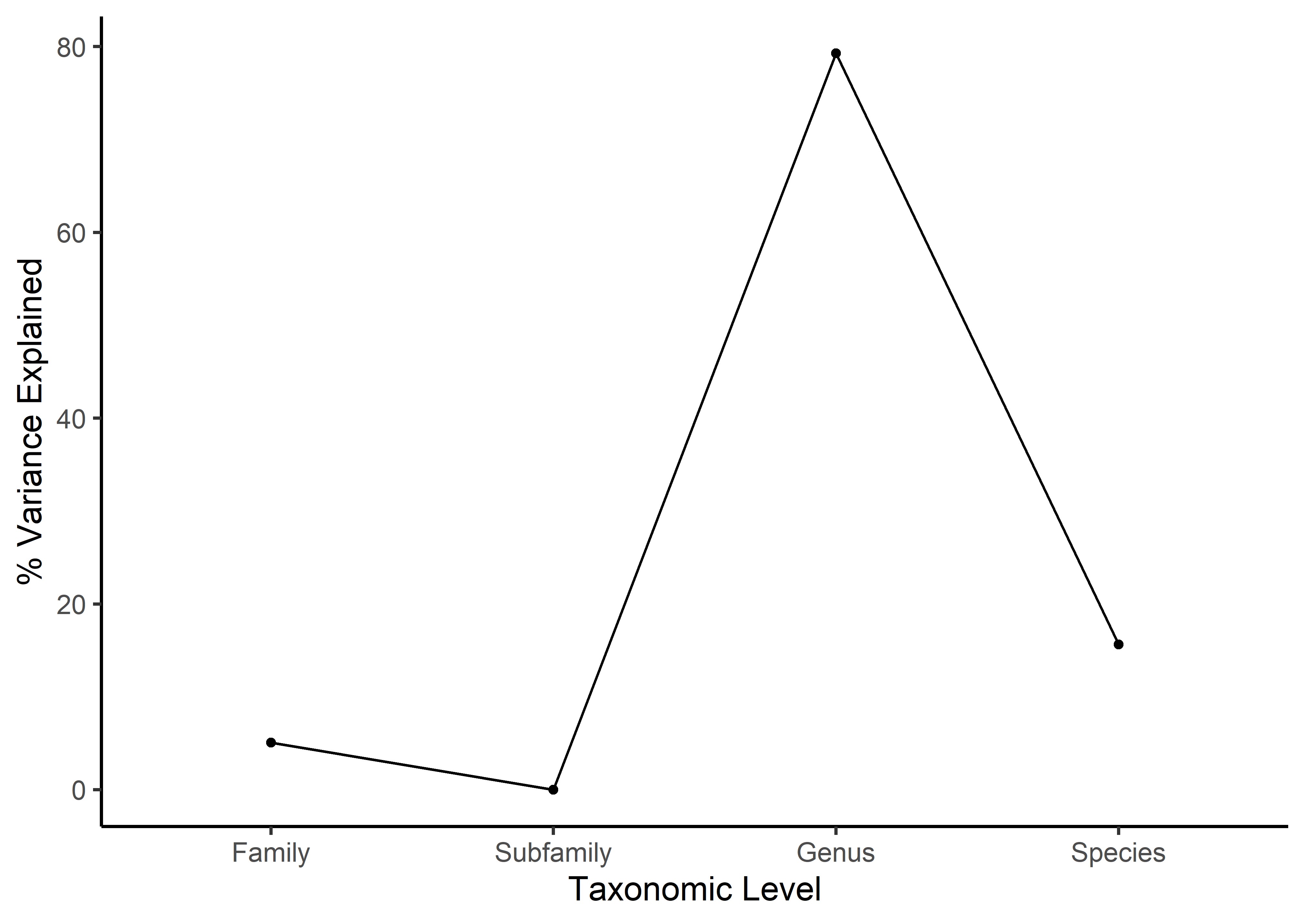

Supplement: nvae110_suppl_Supplementary_Figure_S2 [file nvae110_suppl_supplementary_figure_s2.jpeg]

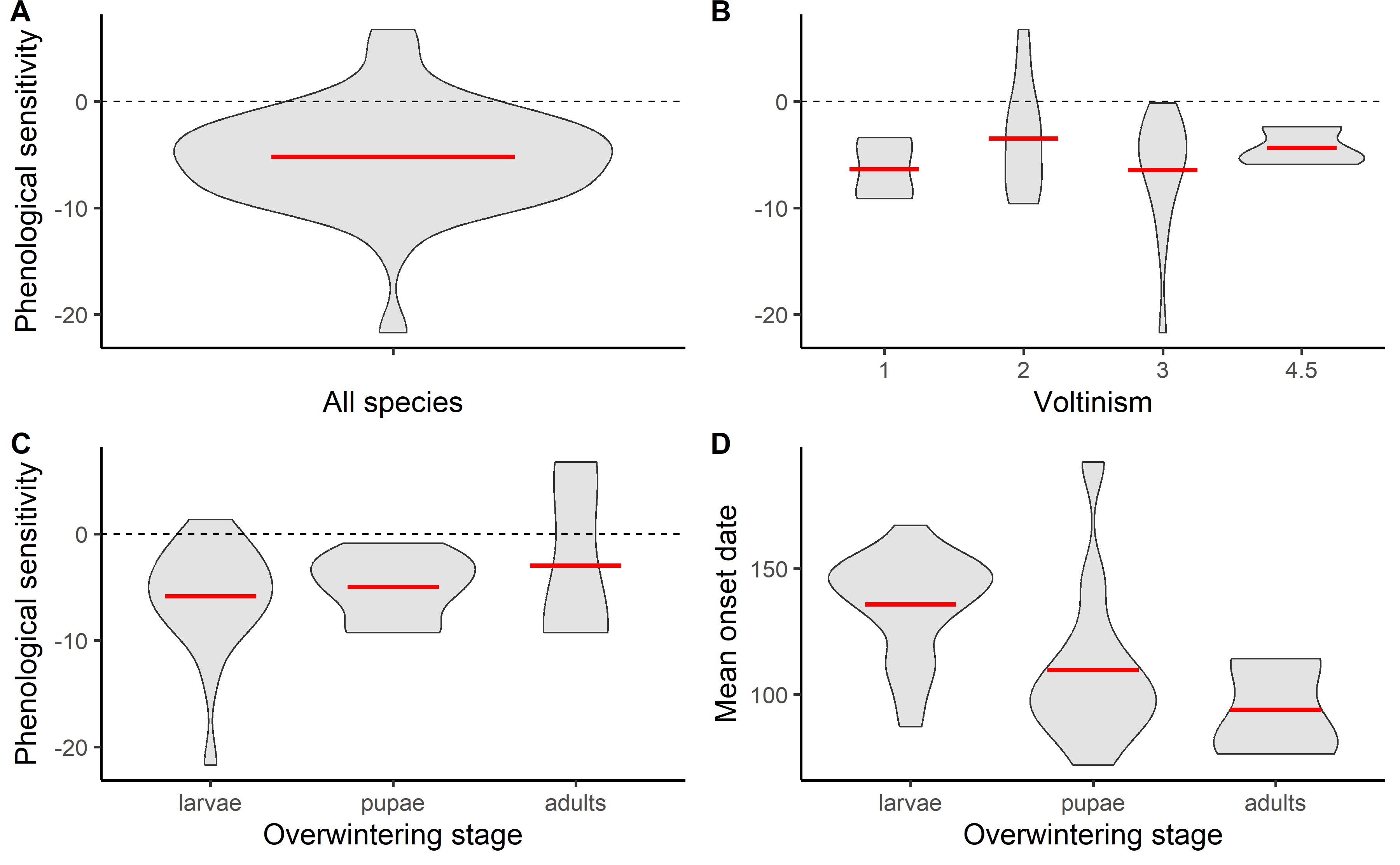

Supplement: nvae110_suppl_Supplementary_Figure_S3 [file nvae110_suppl_supplementary_figure_s3.jpeg]

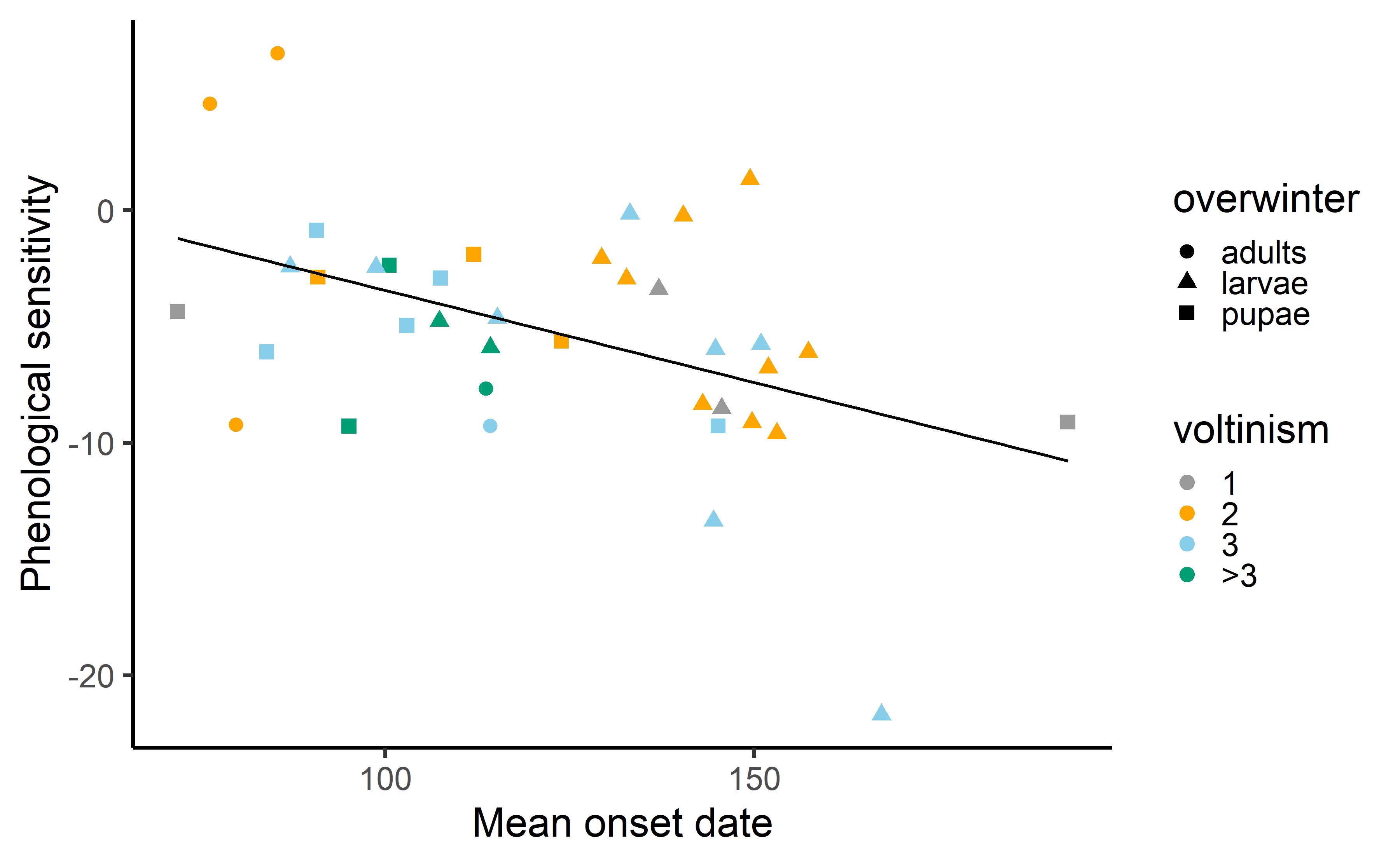

Supplement: nvae110_suppl_Supplementary_Figure_S4 [file nvae110_suppl_supplementary_figure_s4.jpeg]

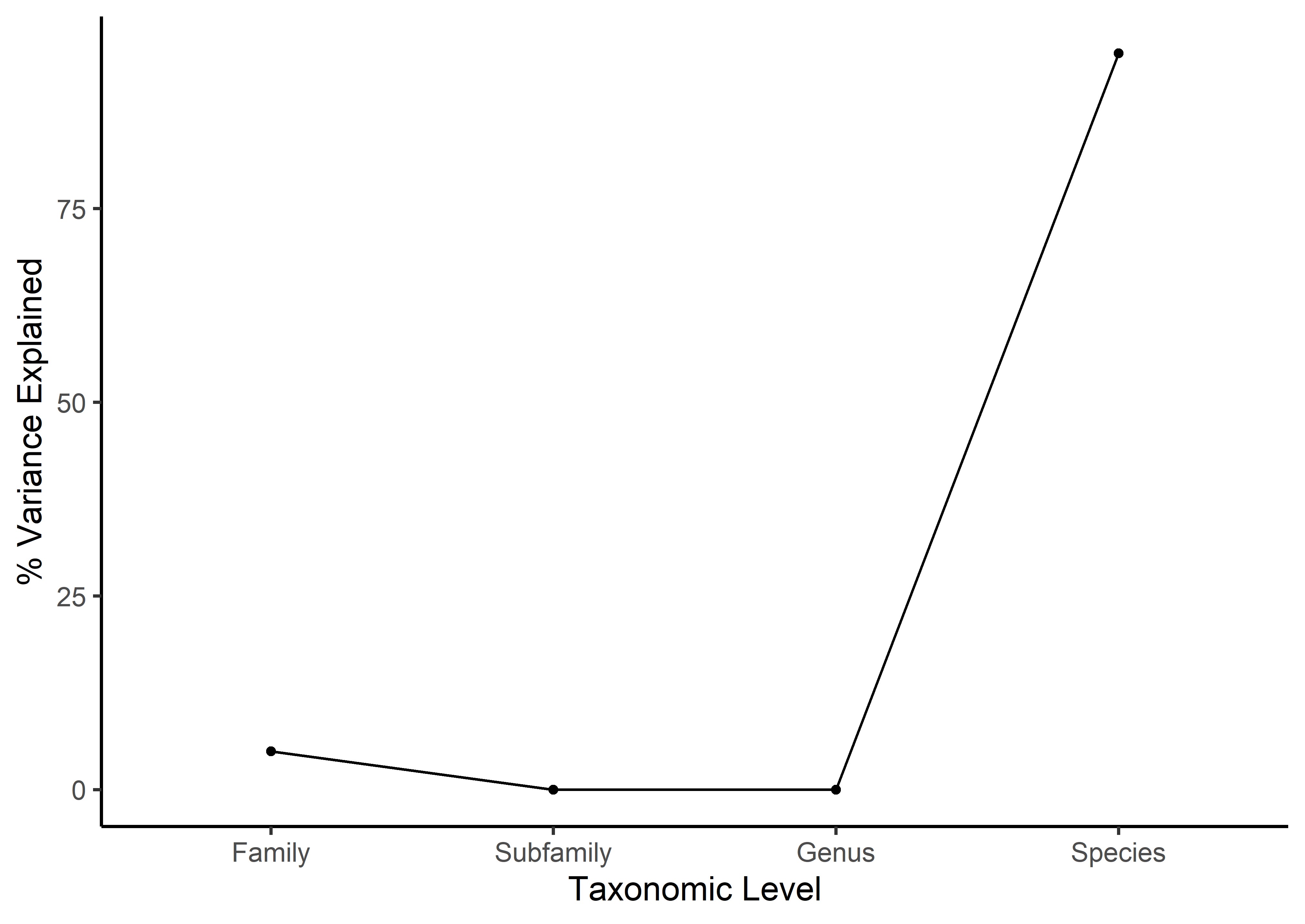

Supplement: nvae110_suppl_Supplementary_Figure_S5 [file nvae110_suppl_supplementary_figure_s5.jpeg]
