# Supplementary material for: High voltinism, late-emerging butterflies are sensitive to interannual variation in spring temperature in North Carolina
Source: Environ Entomol. 2024 Nov 7;54(1):77–85. doi: 10.1093/ee/nvae110 (PMC11837338; doi:10.1093/ee/nvae110)
Supplement: nvae110_suppl_Supplementary_Table_S1 [file nvae110_suppl_supplementary_table_s1.docx]

**Supplemental Table 1.** Summary of species and species traits included in analysis.

| **Species** | **Family** | **Voltinism** | **Overwintering stage** | **Reference** |
| --- | --- | --- | --- | --- |
| *Abaeis nicippe* | Pieridae | 3 | adults | LeGrand and Howard 2022, Florida Museum 2021 |
| *Ancyloxypha numitor* | Hesperiidae | 3 | larvae | LeGrand and Howard 2022 |
| *Anthocharis midea* | Pieridae | 1 | pupae | LeGrand and Howard 2022, Butterflies and Moths of North America (BAMONA) 2022 |
| *Asterocampa celtis* | Nymphalidae | 2 | larvae | LeGrand and Howard 2022, Hall and Butler 2009a |
| *Atalopedes campestris* | Hesperiidae | 3 | larvae | LeGrand and Howard 2022, Burgess 2018 |
| *Battus philenor* | Papilionidae | 3 | pupae | LeGrand and Howard 2022, Illinois Department of Natural Resources 2017 |
| *Calycopis cecrops* | Lycaenidae | 2 | larvae | LeGrand and Howard 2022, Hall and Butler 1999 |
| *Celastrina spp.* | Lycaenidae | 3 | pupae | LeGrand and Howard 2022, BAMONA 2022 |
| **Species** | **Family** | **Voltinism** | **Overwintering stage** | **Reference** |
| *Colias eurytheme* | Pieridae | 4.5 | pupae | LeGrand and Howard 2022, BAMONA 2022 |
| *Cupido comyntas* | Lycaenidae | 4.5 | larvae | LeGrand and Howard 2022, BAMONA 2022 |
| *Cyllopsis gemma* | Nymphalidae | 3 | larvae | LeGrand and Howard 2022, BAMONA 2022 |
| *Epargyreus clarus* | Hesperiidae | 2 | pupae | LeGrand and Howard 2022, Hall 2008 |
| *Erynnis spp.* | Hesperiidae | 3 | larvae | LeGrand and Howard 2022, BAMONA 2022 |
| *Euphyes vestris* | Hesperiidae | 2 | larvae | LeGrand and Howard 2022, BAMONA 2022 |
| *Eurytides marcellus* | Papilionidae | 3 | pupae | LeGrand and Howard 2022, Hall and Butler 1998 |
| *Hermeuptychia sosybius* | Nymphalidae | 3 | larvae | LeGrand and Howard 2022, Tan and Lucky 2016 |
| *Lerema accius* | Hesperiidae | 1 | pupae | LeGrand and Howard 2022, Burgess 2018 |
| *Lethe anthedon* | Nymphalidae | 2 | larvae | LeGrand and Howard 2022, Alabama Butterfly Atlas 2022 |
| **Species** | **Family** | **Voltinism** | **Overwintering stage** | **Reference** |
| *Lethe Appalachia* | Nymphalidae | 2 | larvae | LeGrand and Howard 2022, Alabama Butterfly Atlas 2022 |
| *Libytheana carinenta* | Nymphalidae | 2 | adults | LeGrand and Howard 2022, Hall and Butler 2009b |
| *Limenitis archippus* | Nymphalidae | 3 | larvae | LeGrand and Howard 2022, Wisconsin Pollinators |
| *Limenitis arthemis* | Nymphalidae | 3 | larvae | LeGrand and Howard 2022, Hall and Butler 2009c |
| *Megisto cymela* | Nymphalidae | 1 | larvae | LeGrand and Howard 2022, BAMONA 2022 |
| *Papilio glaucus* | Papilionidae | 2 | pupae | LeGrand and Howard 2022, BAMONA 2022 |
| *Papilio polyxenes* | Papilionidae | 3 | pupae | LeGrand and Howard 2022, BAMONA 2022 |
| *Papilio troilus* | Papilionidae | 2 | pupae | LeGrand and Howard 2022, BAMONA 2022 |
| *Phyciodes tharos* | Nymphalidae | 4.5 | larvae | LeGrand and Howard 2022, Alabama Butterfly Atlas 2022 |
| **Species** | **Family** | **Voltinism** | **Overwintering stage** | **Reference** |
| *Pieris rapae* | Pieridae | 5 | pupae | LeGrand and Howard 2022, BAMONA 2022 |
| *Polites origenes* | Hesperiidae | 2 | larvae | LeGrand and Howard 2022, BAMONA 2022 |
| *Polygonia comma* | Nymphalidae | 2 | adults | LeGrand and Howard 2022 |
| *Polygonia interrogationis* | Nymphalidae | 2 | adults | LeGrand and Howard 2022 |
| *Pompeius verna* | Hesperiidae | 2 | larvae | LeGrand and Howard 2022, Alabama Butterfly Atlas 2022. |
| *Pyrgus communis* | Hesperiidae | 3 | larvae | LeGrand and Howard 2022, BAMONA 2022. Note possible name change |
| *Speyeria cybele* | Nymphalidae | 1 | larvae | LeGrand and Howard 2022, Alabama Butterfly Atlas 2022 |
| *Strymon melinus* | Lycaenidae | 3 | pupae | LeGrand and Howard 2022, BAMONA 2022 |
| *Thorybes daunus* | Hesperiidae | 2 | larvae | LeGrand and Howard 2022, BAMONA 2022 |
| *Vanessa virginiensis* | Nymphalidae | 3.5 | adults | LeGrand and Howard 2022, Hall 2009 |
| **Species** | **Family** | **Voltinism** | **Overwintering stage** | **Reference** |
| *Wallengrenia otho* | Hesperiidae | 2 | larvae | LeGrand and Howard 2022, Burgess 2018. |

**Supplemental Table 1 citations**

Alabama Butterfly Atlas, 2022. Available from <https://alabama.butterflyatlas.usf.edu/species/list>

Butterflies and Moths of North America, 2022. Available from <https://www.butterfliesandmoths.org/>

Burgess L. 2018. Butterflies of South Carolina. Clemson Cooperative Extension Home & Garden Information CenterAvailable from <https://hgic.clemson.edu/factsheet/butterflies-of-south-carolina/>

Florida Museum, 2021. Available from https://www.floridamuseum.ufl.edu/wildflowers/

Hall DW. 2008 Silver-spotted skipper. University of Florida Department of Entomology and Nematology. Available from <https://entnemdept.ufl.edu/creatures/bfly/silver-spotted_skipper.htm>

Hall DW. 2009. American lady, American painted lady. University of Florida Department of Entomology and Nematology.Available from <https://entnemdept.ufl.edu/creatures/bfly/american_lady.htm>

Hall DW.,Butler JF. 1998. Zebra swallowtail. University of Florida Department of Entomology and Nematology. Available from <https://entnemdept.ufl.edu/creatures/bfly/zebra_swallowtail.htm>

Hall DW., Butler JF. 1999a. Red-banded hairstreak. University of Florida Department of Entomology and Nematology. Available from <https://entnemdept.ufl.edu/creatures/bfly/redbanded_hairstreak.htm>

Hall DW., Butler JF. 2009a. Hackberry Emperor. University of Florida Department of Entomology and Nematology. Available from <https://entnemdept.ufl.edu/creatures/bfly/hackberry_emperor.htm>

Hall DW., Butler JF. 2009b. American snout. University of Florida Department of Entomology and Nematology. Available from <https://entnemdept.ufl.edu/creatures/bfly/american_snout.htm>

Hall DW., Butler JF. 2009c. Red-spotted purple. University of Florida Department of Entomology and Nematology. Available from <https://entnemdept.ufl.edu/creatures/bfly/red-spotted_purple.htm>

Illinois Department of Natural Resources. 2017. Pipevine swallowtail. Available from <https://dnr.illinois.gov/education/wildaboutpages/wildaboutinvertebrates/wildaboutbutterflies/family-papilionidae/wambpipevineswallowtail.html#:~:text=The%20pipevine%20swallowtail%20has%20a,be%20seen%20on%20the%20underside>.

LeGrand H, Howard T. 2022. Butterflies of North Carolina: their distribution and abundance. 29th Approximation. Raleigh (North Carolina): North Carolina Biodiversity Project and North Carolina State Parks.

Tan D., Lucky A. 2016. Carolina satyr. University of Florida Department of Entomology and Nematology. Available from <https://entnemdept.ufl.edu/creatures/BFLY/Carolina_satyr.htm>

Wisconsin Pollinators. Available from https://wisconsinpollinators.com/BU/BU_Details.aspx?butterflyid=59
