# Supplementary material for: High voltinism, late-emerging butterflies are sensitive to interannual variation in spring temperature in North Carolina
Source: Environ Entomol. 2024 Nov 7;54(1):77–85. doi: 10.1093/ee/nvae110 (PMC11837338; doi:10.1093/ee/nvae110)
Supplement: nvae110_suppl_Supplementary_Appendix_S1 [file nvae110_suppl_supplementary_appendix_s1.docx]

**APPENDIX S1: Analysis including outliers**

METHODS

Analyses follow the same methods as described in the main manuscript, but outliers are not excluded.

RESULTS

*Phenological response*

Of the 38 focal species, 35 exhibited earlier onset dates in years with warmer springs (Fig. S3A, all onset date vs. temperature plots with outliers highlighted in Appendix S3), with 5 species exhibiting significant (P < 0.05) negative slopes (Table S3). The median response was for a species to appear 5.2 days earlier for every 1℃ increase in average spring temperature, with some species shifting up to nearly 22 days/℃ (Table S3). When examining year as the explanatory variable, just 4 of the 38 focal species exhibited a significant relationship (P < 0.05), all of which were positive (Table S4).

*Species traits*

When examining a model with voltinism alone, we did not observe a significant difference in phenological sensitivity between butterflies of differing voltinism (F_1,36_ = 0.143, R^2^ = -0.024, P = 0.707, Fig. S3B). When we examined the distribution of phenological sensitivity by overwintering stage (Fig. S3C) and compared this to the distribution of mean onset date by overwintering stage, we observed a pattern in which species that overwinter as larvae tend to have later mean onset dates and those that overwinter as adults tend to have earlier mean onset dates (Fig. S3D), such that mean onset date may partially serves as a proxy for overwintering stage. Therefore, we elected to interpret a more parsimonious model which excluded the categorical overwintering stage in favor of the continuous estimate of mean onset date. In addition, adding overwintering stage (F_5,32_  = 2.04, R^2^ = 0.123, P = 0.099) did not improve the model fit when compared to a less complex model with mean onset date, voltinism, and their interactions (F_3,34_  = 3.078, R^2^ = 0.144, P = 0.040; model comparison: ANOVA, F_2,32_ = 1.089, P = 0.559). We also did not observe a significant effect of the interaction between voltinism and overwintering stage on phenological sensitivity (F_2,32_ = 1.110, P = 0.342).

Conversely to the analysis that excluded outliers, we observed no significant effect of the interaction between voltinism and mean onset date on phenological sensitivity (F_1,34_ = 1.312, P = 0.260 Fig. S5). Instead, we observed a significant effect of mean onset date alone on phenological sensitivity (F_1,34_ = 7.752, R^2^ = 0.144, P = 0.040, Fig. S4).

When examining the influence of taxonomic level on phenological sensitivity, we found that 95% of the variance is explained at the level of species (Fig. S5).
