# Supplementary material for: High voltinism, late-emerging butterflies are sensitive to interannual variation in spring temperature in North Carolina
Source: Environ Entomol. 2024 Nov 7;54(1):77–85. doi: 10.1093/ee/nvae110 (PMC11837338; doi:10.1093/ee/nvae110)
Supplement: nvae110_suppl_Supplementary_Appendix_S2 [file nvae110_suppl_supplementary_appendix_s2.pdf]

APPENDIX S2: Regression plots of onset date (the Julian date on which 10% of records for that year were collected) versus average spring temperature for all species included in analyses. Points where Cook's distance was greater than four divided by the total number of data points for each species were excluded from analysis. The slope of each plot was then used as a measure of phenological sensitivity (days/°C) for each species.

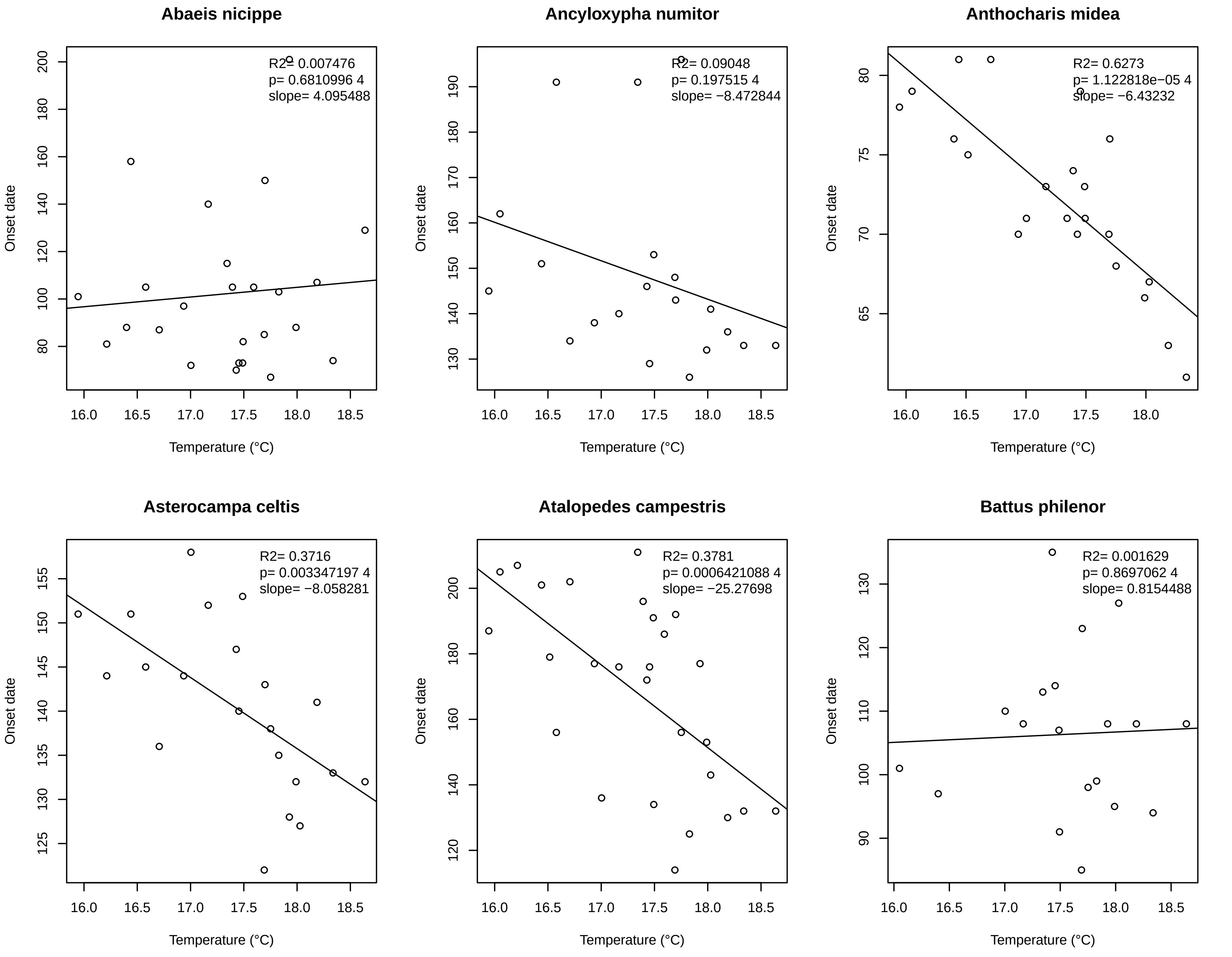
Abaeis nicippe
R2= 0.007476
p= 0.6810996 4
slope= 4.095488
Ancyloxypha numitor
R2= 0.09048
p= 0.197515 4
slope= −8.472844
Anthocharis midea
R2= 0.6273
p= 1.122818e−05 4
slope= −6.43232
Asterocampa celtis
R2= 0.3716
p= 0.003347197 4
slope= −8.058281
Atalopedes campestris
R2= 0.3781
p= 0.0006421088 4
slope= −25.27698
Battus philenor
R2= 0.001629
p= 0.8697062 4
slope= 0.8154488
Onset date
Temperature (°C)

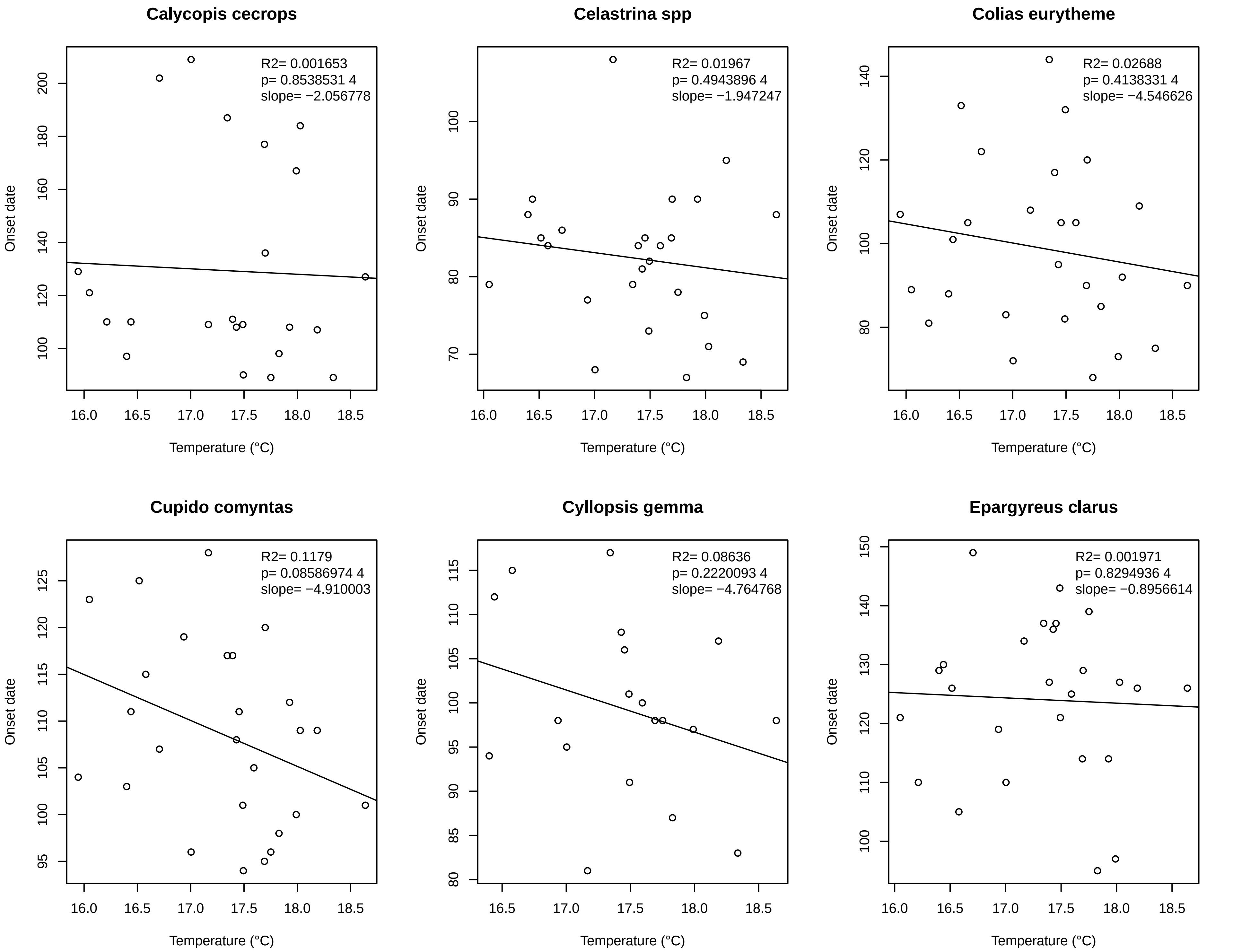
Calycopis cecrops
R2= 0.001653
p= 0.8538531 4
slope= −2.056778
Onset date
Temperature (°C)
Celastrina spp
R2= 0.01967
p= 0.4943896 4
slope= −1.947247
Onset date
Temperature (°C)
Colias eurytheme
R2= 0.02688
p= 0.4138331 4
slope= −4.546626
Onset date
Temperature (°C)
Cupido comyntas
R2= 0.1179
p= 0.08586974 4
slope= −4.910003
Onset date
Temperature (°C)
Cyllopsis gemma
R2= 0.08636
p= 0.2220093 4
slope= −4.764768
Onset date
Temperature (°C)
Epargyreus clarus
R2= 0.001971
p= 0.8294936 4
slope= −0.8956614
Onset date
Temperature (°C)

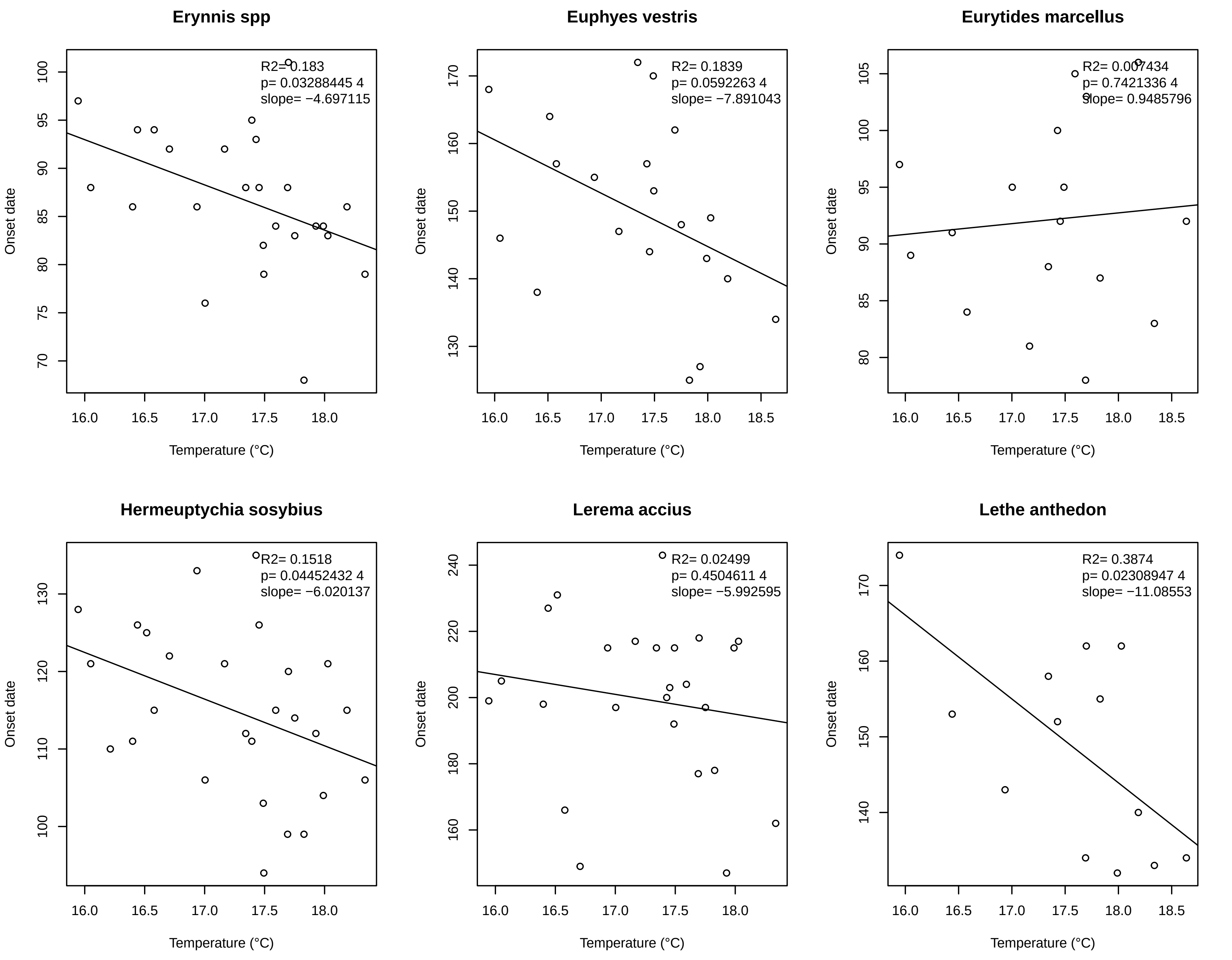
Erynnis spp
R2= 0.183
p= 0.03288445 4
slope= −4.697115
Onset date
Temperature (°C)
Euphyes vestris
R2= 0.1839
p= 0.0592263 4
slope= −7.891043
Onset date
Temperature (°C)
Eurytides marcellus
R2= 0.007434
p= 0.7421336 4
slope= 0.9485796
Onset date
Temperature (°C)
Hermeuptychia sosybius
R2= 0.1518
p= 0.04452432 4
slope= −6.020137
Onset date
Temperature (°C)
Lerema accius
R2= 0.02499
p= 0.4504611 4
slope= −5.992595
Onset date
Temperature (°C)
Lethe anthedon
R2= 0.3874
p= 0.02308947 4
slope= −11.08553
Onset date
Temperature (°C)

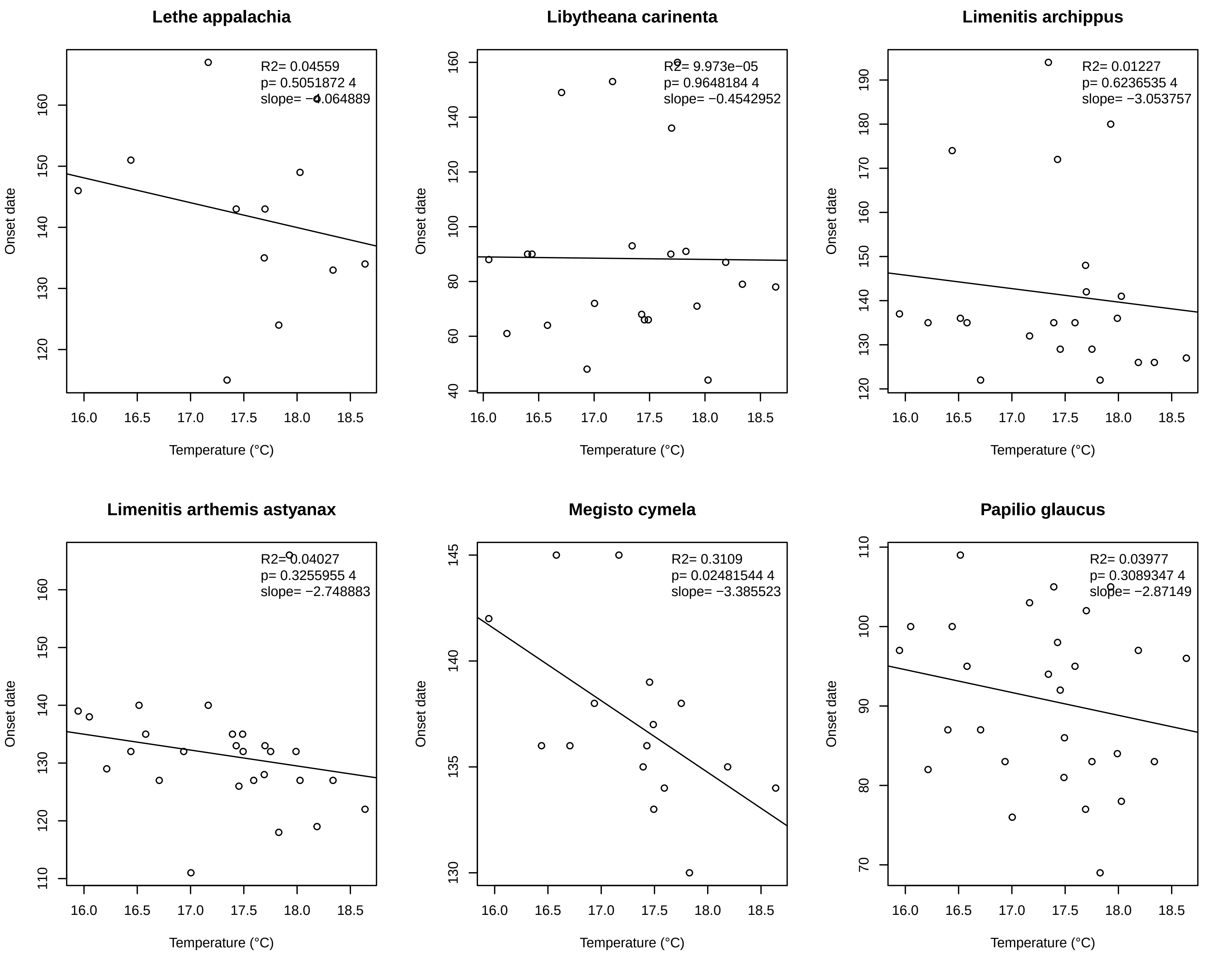
Lethe appalachia
R2= 0.04559
p= 0.5051872 4
slope= −4.064889
Onset date
Temperature (°C)
Libytheana carinenta
R2= 9.973e−05
p= 0.9648184 4
slope= −0.4542952
Onset date
Temperature (°C)
Limenitis archippus
R2= 0.01227
p= 0.6236535 4
slope= −3.053757
Onset date
Temperature (°C)
Limenitis arthemis astyanax
R2= 0.04027
p= 0.3255955 4
slope= −2.748883
Onset date
Temperature (°C)
Megisto cymela
R2= 0.3109
p= 0.02481544 4
slope= −3.385523
Onset date
Temperature (°C)
Papilio glaucus
R2= 0.03977
p= 0.3089347 4
slope= −2.87149
Onset date
Temperature (°C)

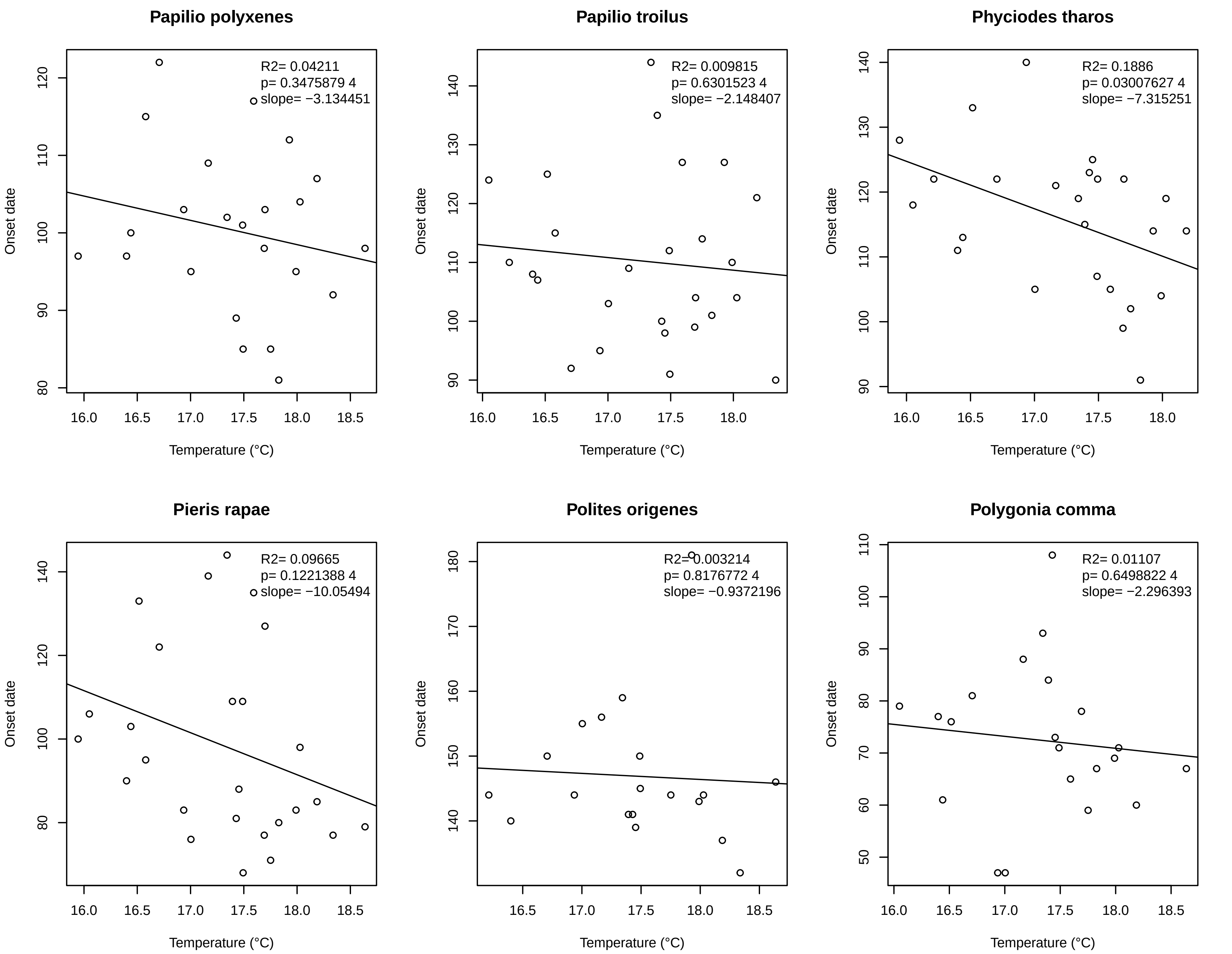
Papilio polyxenes
R2= 0.04211
p= 0.3475879 4
slope= −3.134451
Papilio troilus
R2= 0.009815
p= 0.6301523 4
slope= −2.148407
Phyciodes tharos
R2= 0.1886
p= 0.03007627 4
slope= −7.315251
Pieris rapae
R2= 0.09665
p= 0.1221388 4
slope= −10.05494
Polites origenes
R2= 0.003214
p= 0.8176772 4
slope= −0.9372196
Polygonia comma
R2= 0.01107
p= 0.6498822 4
slope= −2.296393
Onset date
Temperature (°C)

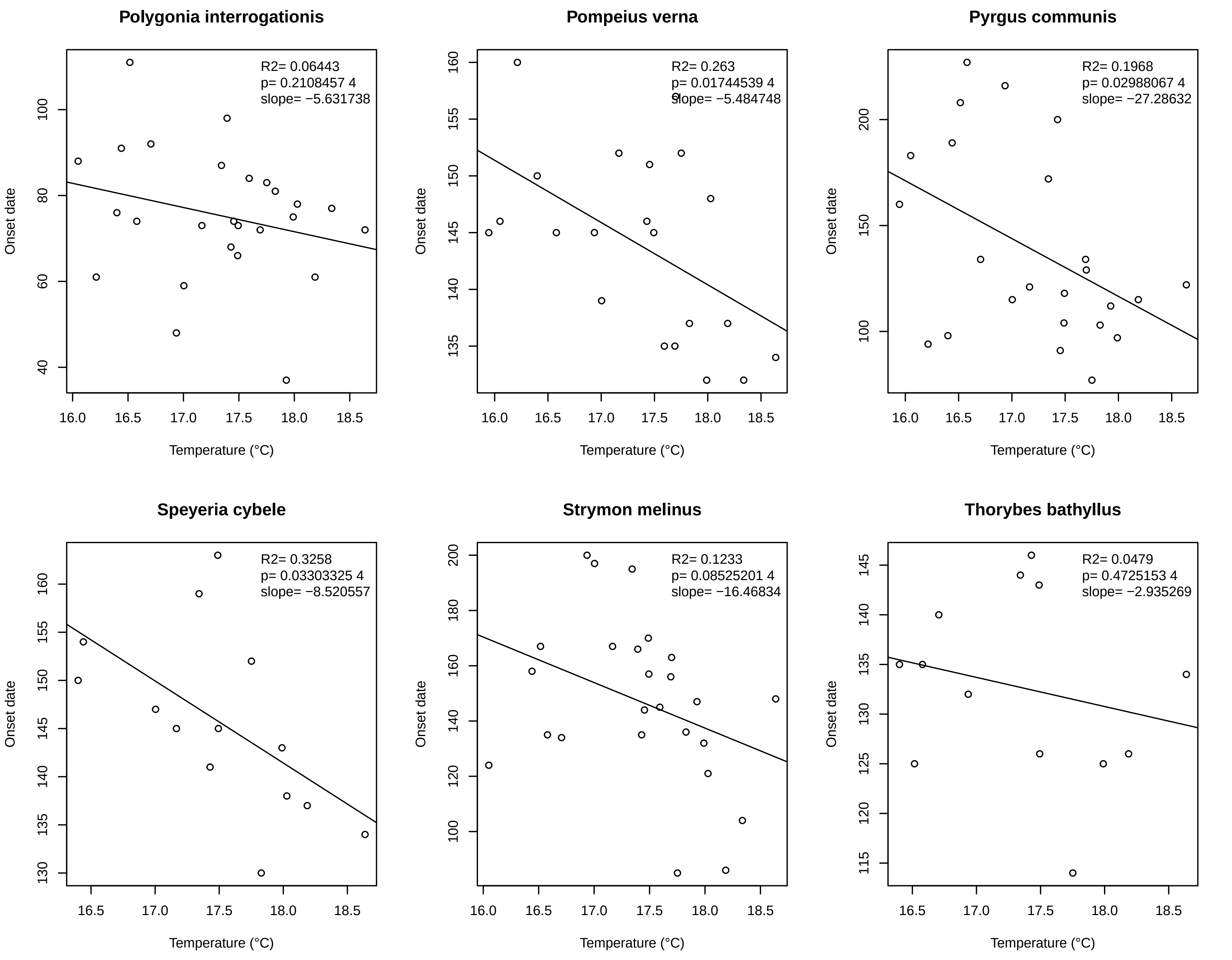
Polygonia interrogationis
R2= 0.06443
p= 0.2108457 4
slope= −5.631738
Pompeius verna
R2= 0.263
p= 0.01744539 4
slope= −5.484748
Pyrgus communis
R2= 0.1968
p= 0.02988067 4
slope= −27.28632
Speyeria cybele
R2= 0.3258
p= 0.03303325 4
slope= −8.520557
Strymon melinus
R2= 0.1233
p= 0.08525201 4
slope= −16.46834
Thorybes bathyllus
R2= 0.0479
p= 0.4725153 4
slope= −2.935269
Onset date
Temperature (°C)

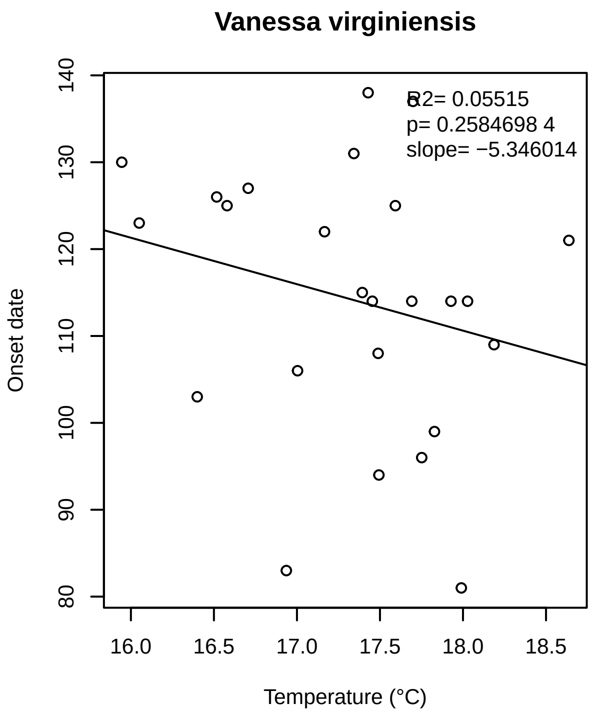
Vanessa virginiensis
R2= 0.05515
p= 0.2584698 4
slope= −5.346014
Onset date
Temperature (°C)

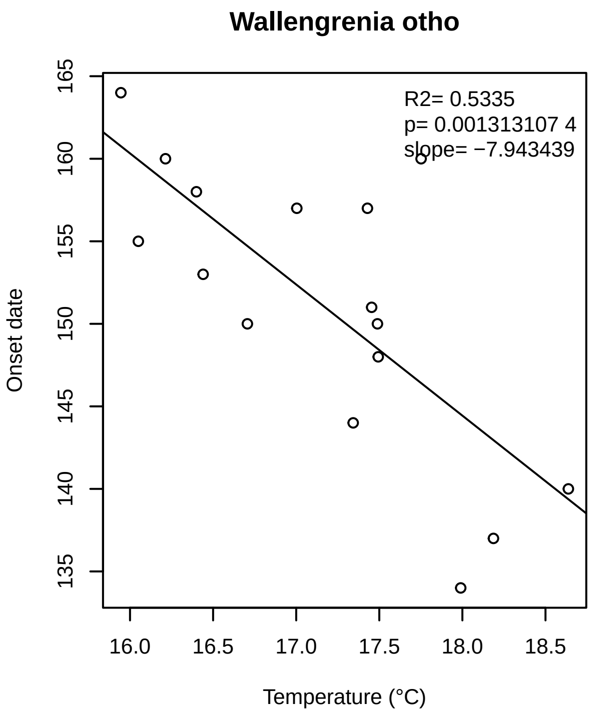
Wallengrenia otho
R2= 0.5335
p= 0.001313107 4
slope= −7.943439
Onset date
Temperature (°C)
