# Supplementary material for: High voltinism, late-emerging butterflies are sensitive to interannual variation in spring temperature in North Carolina
Source: Environ Entomol. 2024 Nov 7;54(1):77–85. doi: 10.1093/ee/nvae110 (PMC11837338; doi:10.1093/ee/nvae110)
Supplement: nvae110_suppl_Supplementary_Table_S2 [file nvae110_suppl_supplementary_table_s2.docx]

**Supplemental Table 2.** Summary of slopes, r-squared values, and p-values from linear regression models of onset date vs. year in NC Triangle butterfly species.

| **Species** | **Slope** | **R-squared** | **P value** |
| --- | --- | --- | --- |
| *Abaeis nicippe* | 1.38 | 0.08 | 0.18 |
| *Ancyloxypha numitor* | 0.24 | 0.02 | 0.55 |
| *Anthocharis midea* | -0.002 | <0.01 | 0.99 |
| *Asterocampa celtis* | -0.21 | 0.03 | 0.48 |
| *Atalopedes campestris* | -0.79 | 0.05 | 0.27 |
| *Battus philenor* | 0.51 | 0.08 | 0.24 |
| *Calycopis cecrops* | 0.05 | <0.01 | 0.97 |
| *Celastrina spp.* | 0.14 | 0.02 | 0.53 |
| *Colias eurytheme* | 0.02 | <0.01 | 0.97 |
| *Cupido comyntas* | -0.07 | <0.01 | 0.78 |
| *Cyllopsis gemma* | -0.09 | <0.01 | 0.77 |
| *Epargyreus clarus* | 0.22 | 0.02 | 0.53 |
| *Erynnis spp.* | 0.08 | 0.01 | 0.62 |
| *Euphyes vestris* | 0.07 | <0.01 | 0.89 |
| *Eurytides marcellus* | -0.67 | 0.21 | 0.07 |
| *Hermeuptychia sosybius* | -0.13 | 0.01 | 0.59 |
| *Lerema accius* | 0.19 | <0.01 | 0.79 |
| *Lethe anthedon* | 1.05 | 0.20 | 0.13 |
| *Lethe appalachia* | -1.12 | 0.12 | 0.36 |
| *Libytheana carinenta* | 0.51 | 0.02 | 0.55 |
| *Limenitis archippus* | 0.69 | 0.13 | 0.10 |
| *Limenitis arthemis* | 0.35 | 0.06 | 0.23 |
| *Megisto cymela* | 0.06 | 0.03 | 0.58 |
| *Papilio glaucus* | 0.08 | 0.01 | 0.74 |
| *Papilio polyxenes* | -0.35 | 0.06 | 0.27 |
| *Papilio troilus* | -0.15 | 0.01 | 0.67 |
| *Phyciodes tharos* | -0.14 | 0.01 | 0.63 |
| *Pieris rapae* | -0.18 | 0.01 | 0.75 |
| *Polites origenes* | 0.48 | 0.24 | 0.04 |
| *Polygonia comma* | 0.75 | 0.10 | 0.13 |
| *Polygonia interrogationis* | -0.14 | 0.01 | 0.68 |
| *Pompeius verna* | -0.15 | 0.02 | 0.55 |
| *Pyrgus communis* | 0.96 | 0.02 | 0.46 |
| *Speyeria cybele* | -0.14 | 0.01 | 0.77 |
| *Strymon melinus* | 1.16 | 0.08 | 0.13 |
| *Thorybes daunus* | 0.92 | 0.37 | 0.04 |
| *Vanessa virginiensis* | 0.33 | 0.03 | 0.42 |
| *Wallengrenia otho* | -0.19 | 0.02 | 0.62 |
