# Supplementary material for: High voltinism, late-emerging butterflies are sensitive to interannual variation in spring temperature in North Carolina
Source: Environ Entomol. 2024 Nov 7;54(1):77–85. doi: 10.1093/ee/nvae110 (PMC11837338; doi:10.1093/ee/nvae110)
Supplement: nvae110_suppl_Supplementary_Table_S3 [file nvae110_suppl_supplementary_table_s3.docx]

**Supplemental Table 3.** Summary of slopes, r-squared values, p-values, and sample sizes per species from linear regression models of onset date vs. spring temperature in NC Triangle butterfly species, in addition to the mean and standard deviation of onset date for each species, with outliers included in analysis.

| **Species** | **Slope** | **R-squared** | **P value** | **Mean onset date** | **Standard deviation onset date** | **Number of records** |
| --- | --- | --- | --- | --- | --- | --- |
| *Abaeis nicippe* | -9.26 | 0.02 | 0.47 | 114.21 | 46.72 | 1362 |
| *Ancyloxypha numitor* | -5.74 | 0.03 | 0.43 | 150.90 | 23.46 | 563 |
| *Anthocharis midea* | -4.36 | 0.24 | 0.01 | 71.84 | 6.47 | 448 |
| *Asterocampa celtis* | -8.34 | 0.16 | 0.06 | 143.00 | 14.76 | 414 |
| *Atalopedes campestris* | -21.69 | 0.28 | <0.01 | 167.21 | 29.26 | 1176 |
| *Battus philenor* | -2.91 | 0.02 | 0.51 | 107.45 | 13.20 | 458 |
| *Calycopis cecrops* | -2.06 | <0.01 | 0.85 | 129.30 | 38.08 | 577 |
| *Celastrina spp.* | -6.09 | 0.09 | 0.12 | 83.93 | 14.64 | 1139 |
| *Colias eurytheme* | -2.36 | <0.01 | 0.69 | 100.54 | 21.37 | 922 |
| *Cupido comyntas* | -4.75 | 0.10 | 0.09 | 17.32 | 10.60 | 1728 |
| *Cyllopsis gemma* | -2.44 | 0.03 | 0.50 | 98.75 | 9.96 | 405 |
| *Epargyreus clarus* | -5.63 | 0.07 | 0.17 | 123.86 | 15.12 | 1431 |
| *Erynnis spp.* | -2.42 | 0.04 | 0.29 | 87.07 | 8.47 | 1274 |
| *Euphyes vestris* | -6.10 | 0.04 | 0.37 | 157.35 | 23.41 | 392 |
| *Eurytides marcellus* | -0.85 | <0.01 | 0.80 | 90.67 | 10.15 | 282 |
| *Hermeuptychia sosybius* | -4.63 | 0.10 | 0.10 | 115.18 | 10.37 | 1119 |
| *Lerema accius* | -9.11 | 0.04 | 0.28 | 192.50 | 30.98 | 1012 |
| *Lethe anthedon* | -9.59 | 0.11 | 0.25 | 153.07 | 21.17 | 207 |
| *Lethe appalachia* | -0.22 | <0.001 | 0.97 | 140.38 | 14.92 | 213 |
| *Libytheana carinenta* | 6.77 | 0.02 | 0.50 | 85.39 | 34.96 | 550 |
| *Limenitis archippus* | -5.96 | 0.03 | 0.44 | 144.74 | 24.94 | 421 |
| *Limenitis arthemis* | -0.15 | <0.001 | 0.98 | 133.14 | 19.70 | 1161 |
| *Megisto cymela* | -3.39 | 0.31 | 0.02 | 137.06 | 4.11 | 241 |
| *Papilio glaucus* | -2.87 | 0.04 | 0.31 | 90.86 | 10.33 | 2205 |
| *Papilio polyxenes* | -4.95 | 0.07 | 0.22 | 102.92 | 13.71 | 580 |
| **Species** | **Slope** | **R-squared** | **P value** | **Mean onset date** | **Standard deviation onset date** | **Number of records** |
| *Papilio troilus* | -1.89 | <0.01 | 0.65 | 112.00 | 14.94 | 824 |
| *Phyciodes tharos* | -5.90 | 0.12 | 0.07 | 114.25 | 12.26 | 1967 |
| *Pieris rapae* | -9.27 | 0.07 | 0.18 | 95.07 | 25.35 | 1092 |
| *Polites origenes* | 1.35 | <0.01 | 0.82 | 149.40 | 15.24 | 343 |
| *Polygonia comma* | 4.58 | 0.02 | 0.49 | 76.24 | 22.80 | 549 |
| *Polygonia interrogationis* | -9.22 | 0.09 | 0.12 | 79.79 | 21.97 | 982 |
| *Pompeius verna* | -6.76 | 0.05 | 0.30 | 151.92 | 22.93 | 454 |
| *Pyrgus communis* | -13.35 | 0.04 | 0.31 | 144.46 | 47.88 | 714 |
| *Speyeria cybele* | -8.52 | 0.33 | 0.03 | 145.57 | 9.42 | 217 |
| *Strymon melinus* | -9.27 | 0.04 | 0.30 | 145.11 | 32.96 | 726 |
| *Thorybes bathyllus* | -2.94 | 0.05 | 0.47 | 132.69 | 9.29 | 175 |
| *Vanessa virginiensis* | -7.67 | 0.09 | 0.12 | 113.64 | 18.37 | 1437 |
| *Wallengrenia otho* | -9.12 | 0.50 | <0.01 | 149.71 | 10.26 | 240 |
