# Supplementary material for: High voltinism, late-emerging butterflies are sensitive to interannual variation in spring temperature in North Carolina
Source: Environ Entomol. 2024 Nov 7;54(1):77–85. doi: 10.1093/ee/nvae110 (PMC11837338; doi:10.1093/ee/nvae110)
Supplement: nvae110_suppl_Supplementary_Appendix_S3 [file nvae110_suppl_supplementary_appendix_s3.pdf]

APPENDIX S3: Regression plots of onset date (the Julian date on which 10% of records for that year were collected) versus average spring temperature for all species included in analyses, with outliers included. Outliers were identified as each point where Cook's distance was greater than four divided by the total number of data points. These points are indicated with red circles. The slope of each plot was then used as a measure of phenological sensitivity (days/°C) for each species for analyses that included outliers.

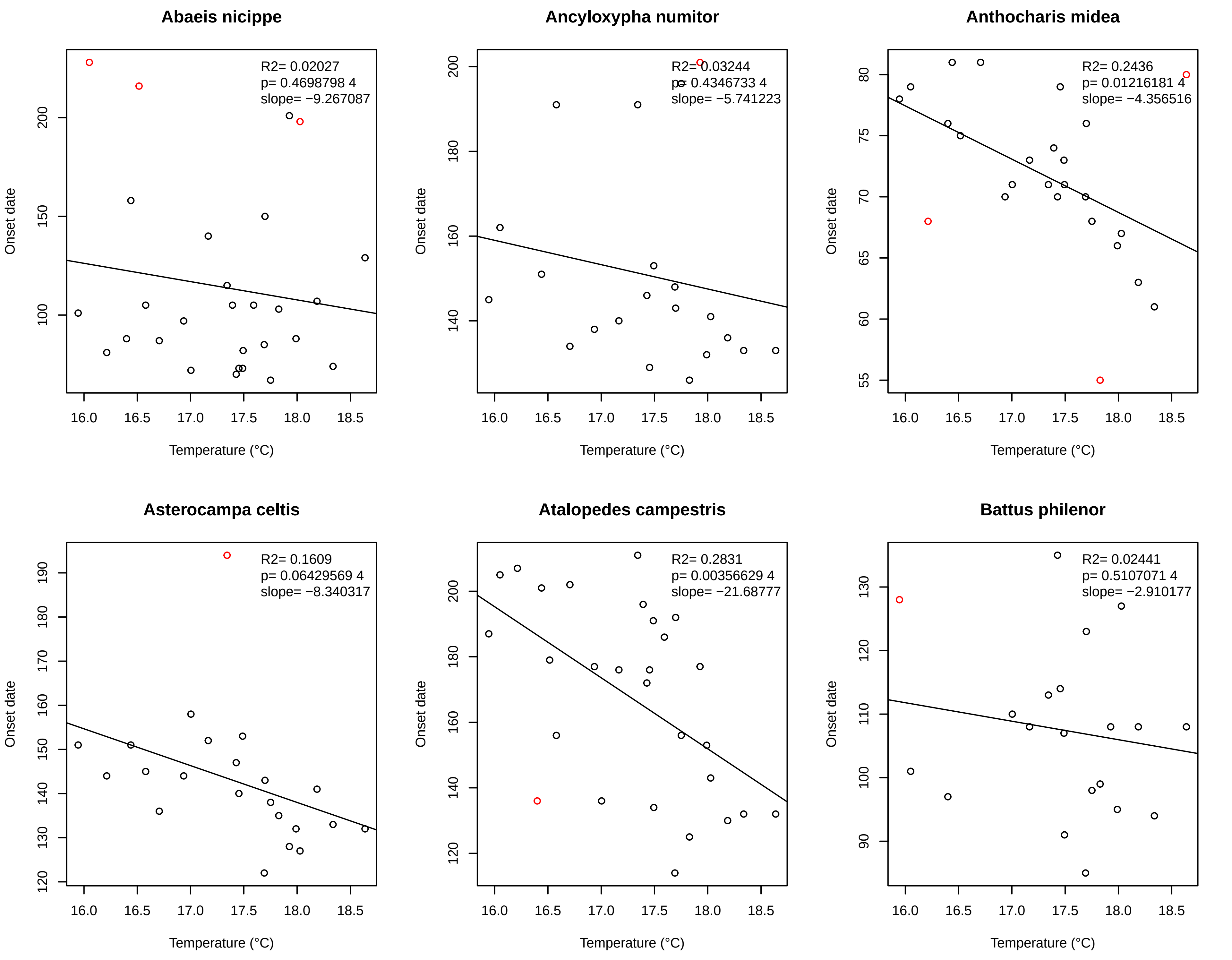
Abaeis nicippe
R2= 0.02027
p= 0.4698798 4
slope= −9.267087
Onset date
Temperature (°C)
Ancyloxypha numitor
R2= 0.03244
p= 0.4346733 4
slope= −5.741223
Onset date
Temperature (°C)
Anthocharis midea
R2= 0.2436
p= 0.01216181 4
slope= −4.356516
Onset date
Temperature (°C)
Asterocampa celtis
R2= 0.1609
p= 0.06429569 4
slope= −8.340317
Onset date
Temperature (°C)
Atalopedes campestris
R2= 0.2831
p= 0.00356629 4
slope= −21.68777
Onset date
Temperature (°C)
Battus philenor
R2= 0.02441
p= 0.5107071 4
slope= −2.910177
Onset date
Temperature (°C)

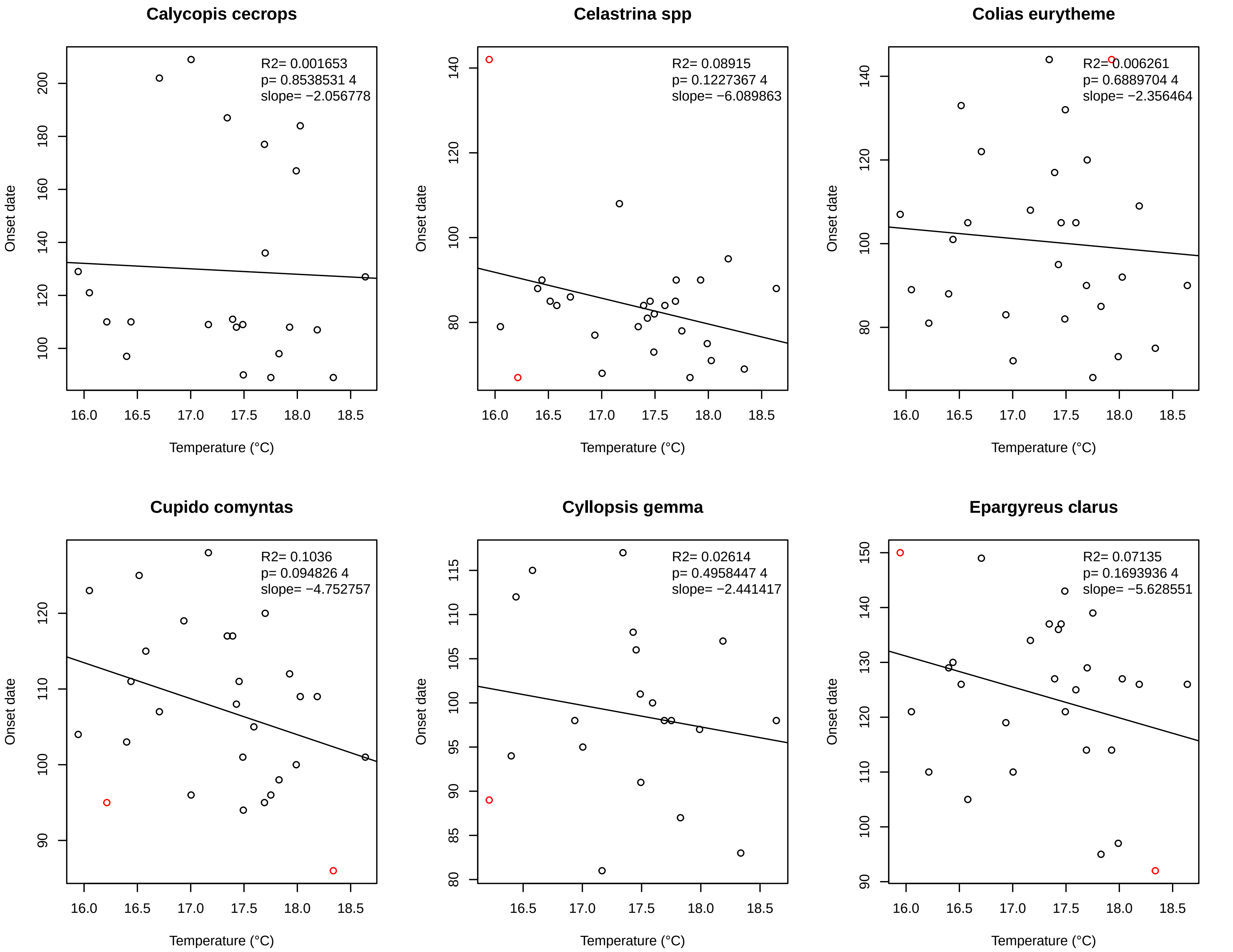

Calycopis cecrops
R2= 0.001653
p= 0.8538531 4
slope= −2.056778
Celastrina spp
R2= 0.08915
p= 0.1227367 4
slope= −6.089863
Colias eurytheme
R2= 0.006261
p= 0.6889704 4
slope= −2.356464
Cupido comyntas
R2= 0.1036
p= 0.094826 4
slope= −4.752757
Cyllopsis gemma
R2= 0.02614
p= 0.4958447 4
slope= −2.441417
Epargyreus clarus
R2= 0.07135
p= 0.1693936 4
slope= −5.628551
Onset date
Temperature (°C)

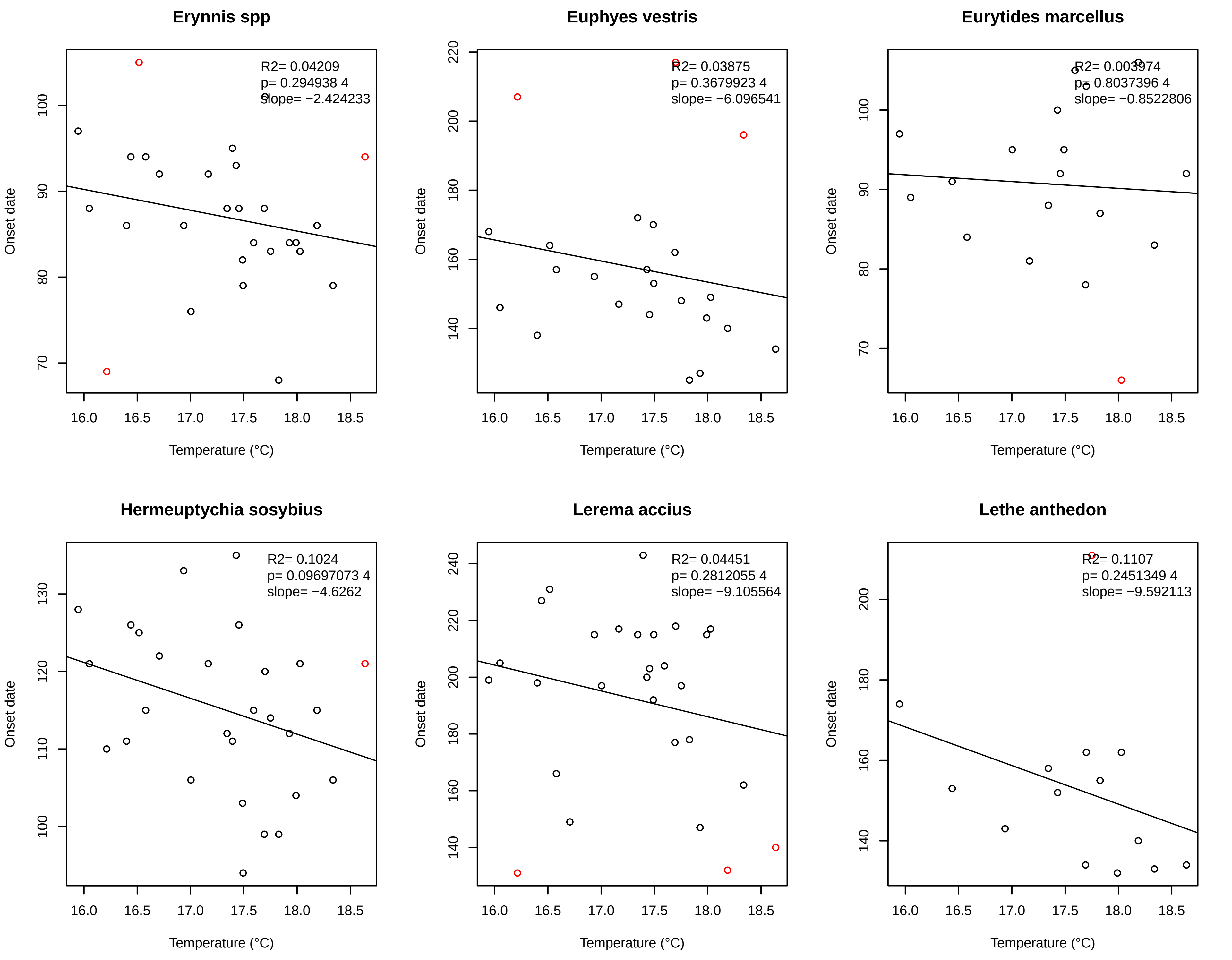

Erynnis spp
R2= 0.04209
p= 0.294938 4
slope= −2.424233
Euphyes vestris
R2= 0.03875
p= 0.3679923 4
slope= −6.096541
Eurytides marcellus
R2= 0.003974
p= 0.8037396 4
slope= −0.8522806
Hermeuptychia sosybius
R2= 0.1024
p= 0.09697073 4
slope= −4.6262
Lerema accius
R2= 0.04451
p= 0.2812055 4
slope= −9.105564
Lethe anthedon
R2= 0.1107
p= 0.2451349 4
slope= −9.592113
Onset date
Temperature (°C)

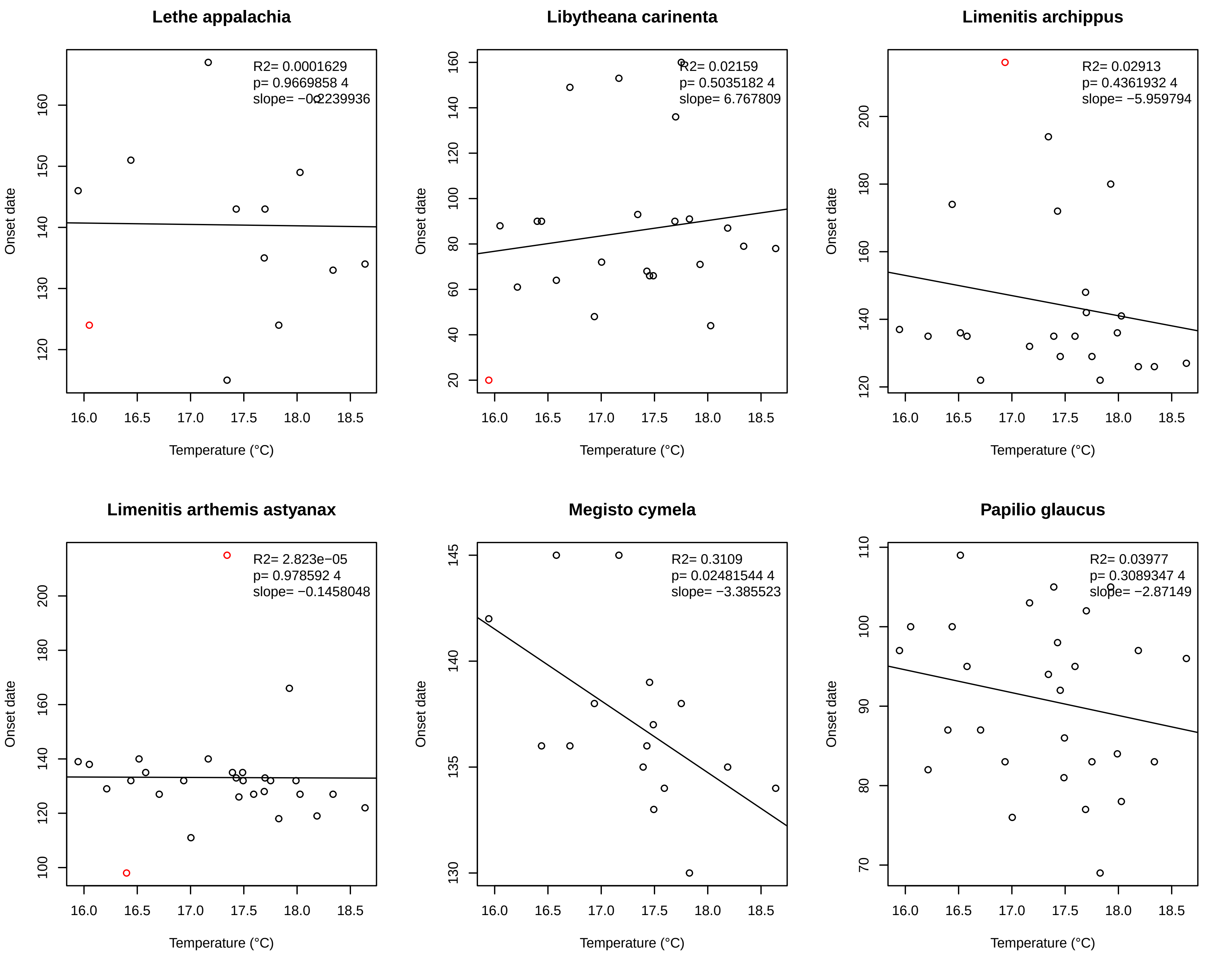
Lethe appalachia
R2= 0.0001629
p= 0.9669858 4
slope= −0.2239936
Onset date
Temperature (°C)
Libytheana carinenta
R2= 0.02159
p= 0.5035182 4
slope= 6.767809
Onset date
Temperature (°C)
Limenitis archippus
R2= 0.02913
p= 0.4361932 4
slope= −5.959794
Onset date
Temperature (°C)
Limenitis arthemis astyanax
R2= 2.823e−05
p= 0.978592 4
slope= −0.1458048
Onset date
Temperature (°C)
Megisto cymela
R2= 0.3109
p= 0.02481544 4
slope= −3.385523
Onset date
Temperature (°C)
Papilio glaucus
R2= 0.03977
p= 0.3089347 4
slope= −2.87149
Onset date
Temperature (°C)

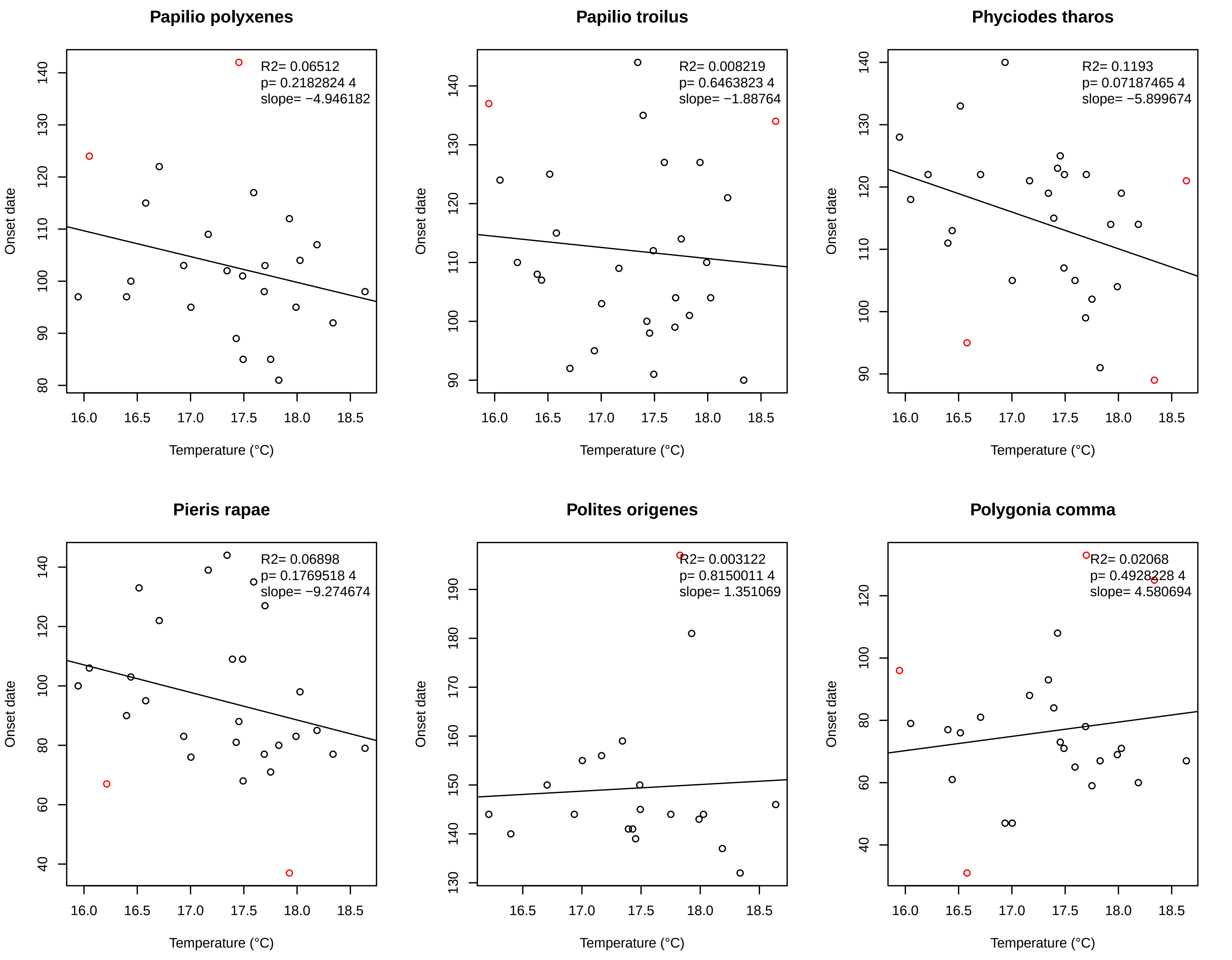

Papilio polyxenes
R2= 0.06512
p= 0.2182824 4
slope= −4.946182
Papilio troilus
R2= 0.008219
p= 0.6463823 4
slope= −1.88764
Phyciodes tharos
R2= 0.1193
p= 0.07187465 4
slope= −5.899674
Pieris rapae
R2= 0.06898
p= 0.1769518 4
slope= −9.274674
Polites origenes
R2= 0.003122
p= 0.8150011 4
slope= 1.351069
Polygonia comma
R2= 0.02068
p= 0.4928228 4
slope= 4.580694
Onset date
Temperature (°C)

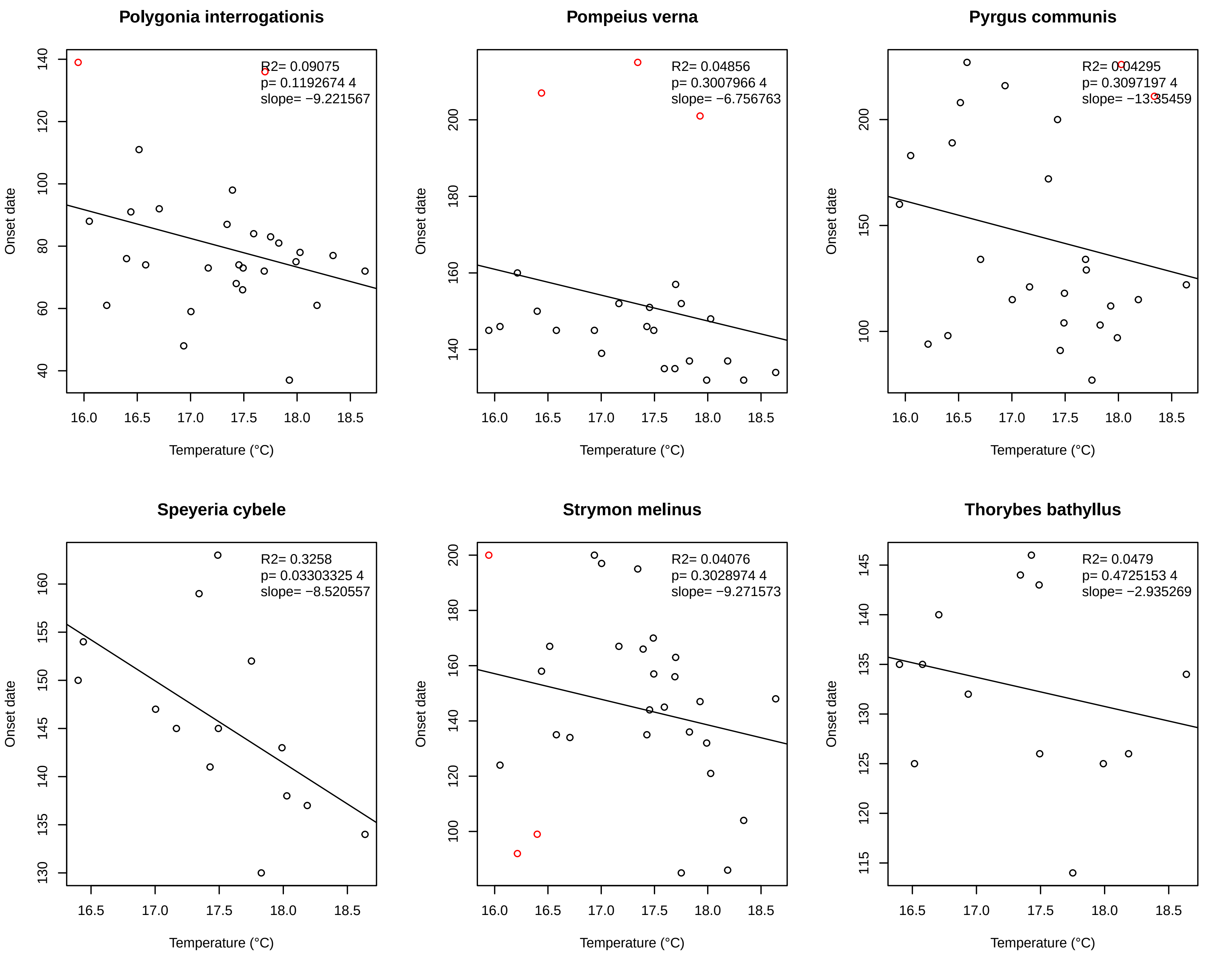
Polygonia interrogationis
R2= 0.09075
p= 0.1192674 4
slope= −9.221567
Pompeius verna
R2= 0.04856
p= 0.3007966 4
slope= −6.756763
Pyrgus communis
R2= 0.04295
p= 0.3097197 4
slope= −13.35459
Speyeria cybele
R2= 0.3258
p= 0.03303325 4
slope= −8.520557
Strymon melinus
R2= 0.04076
p= 0.3028974 4
slope= −9.271573
Thorybes bathyllus
R2= 0.0479
p= 0.4725153 4
slope= −2.935269
Onset date
Temperature (°C)

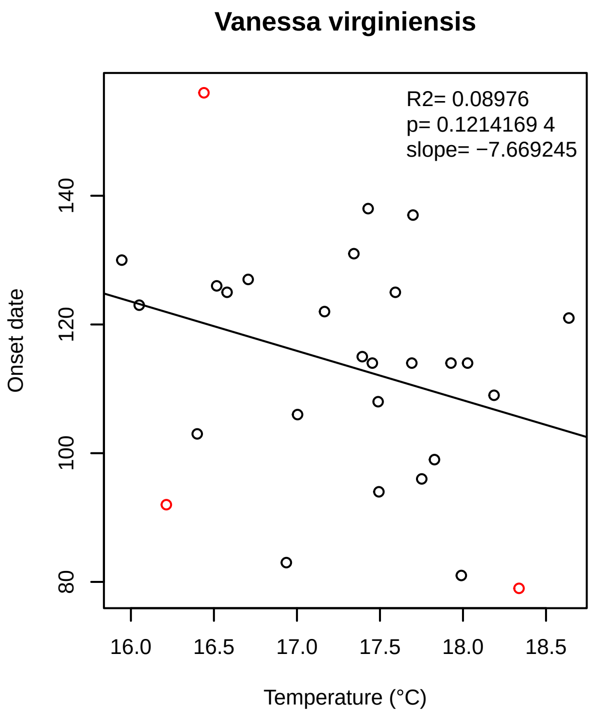
Vanessa virginiensis
R2= 0.08976
p= 0.1214169 4
slope= −7.669245
Onset date
80
100
120
140
16.0
16.5
17.0
17.5
18.0
18.5
Temperature (°C)

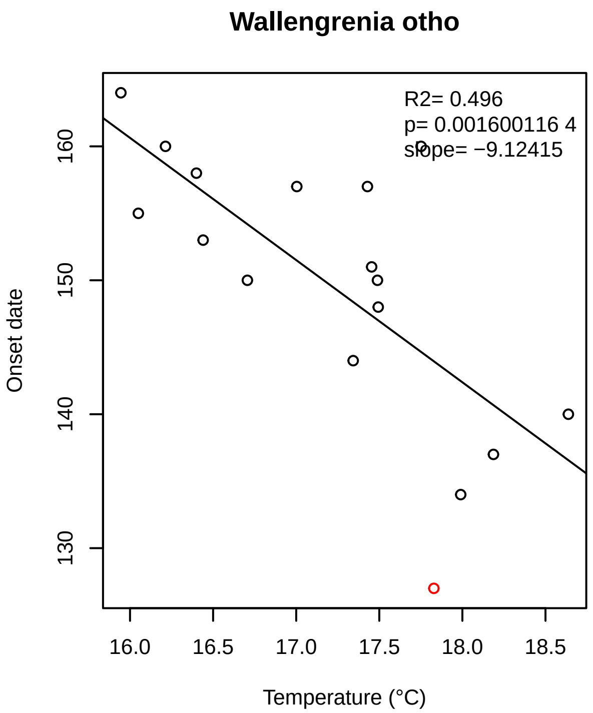
Wallengrenia otho
R2= 0.496
p= 0.001600116 4
slope= −9.12415
Onset date
130
140
150
160
16.0
16.5
17.0
17.5
18.0
18.5
Temperature (°C)
